# Automated Assignment of ^15^N And ^13^C Enrichment Levels in Doubly-Labeled Proteins

**DOI:** 10.1021/jasms.4c00218

**Published:** 2024-08-30

**Authors:** Elijah T. Roberts, Alexander R. Davis, Jeremy T. Risher, Adam W. Barb, I. Jonathan Amster

**Affiliations:** † Department of Chemistry, University of Georgia, Athens, Georgia 30602, United States; ‡ Department of Biochemistry and Molecular Biology, University of Georgia, Athens, Georgia 30602, United States; § Complex Carbohydrate Research Center, University of Georgia, Athens, Georgia 30602, United States

## Abstract

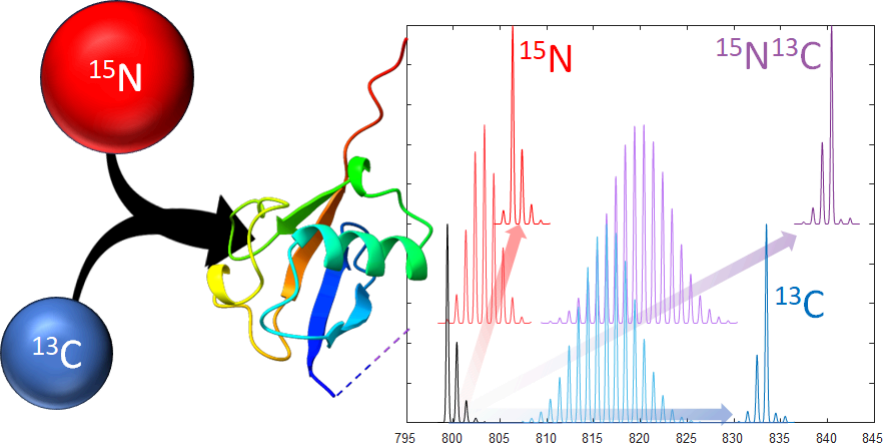

Uniform enrichment of ^15^N and ^13^C in proteins is commonly employed for 2D heteronuclear NMR measurements of the three-dimensional protein structure. Achieving a high degree of enrichment of both elements is important for obtaining high quality data. Uniform labeling of proteins and glycoproteins expressed in higher organisms (yeast or mammalian cell lines) is more challenging than expression in *Escherichia coli*, a prokaryote that grows on simple, chemically defined media but does not provide appropriate eukaryotic modifications. It is difficult to achieve complete incorporation of both heavy isotopes, and quality control measures are important for quantitating the level of their enrichment. Mass spectrometry measurements of the isotopic distribution of the intact protein or its proteolytic fragments provide the means to assess the enrichment level. A mass accuracy of 1 ppm or better is shown to be required to distinguish the correct combination of ^13^C and ^15^N enrichment due to subtle shifts in peak centroids with differences in the underlying, but unresolved, isotopic fine structure. A simple computer program was developed to optimize the fitting of experimental isotope patterns to statistically derived distributions. This method can determine the isotopic abundance from isotope patterns and isotopologue masses in conventional MS data for peptides, intact proteins, and glycans. For this purpose, MATLAB’s isotope simulator, isotopicdist, has been modified to permit the variation of ^15^N and ^13^C enrichment levels and to perform a two-dimensional grid search of enrichment levels of both isotopes. We also incorporated an alternate isotope simulator, js-emass, into MATLAB for use in the same fitting program. Herein we benchmark this technique on natural abundance ubiquitin and uniformly [^15^N,^13^C]-labeled ubiquitin at both the intact and peptide level, outline considerations for data quality and mass accuracy, and report several improvements we have made to the previously reported analysis of the [^15^N,^13^C]-enriched human IgG Fc domain, a glycoprotein that has been expressed in *Saccharomyces cerevisiae*.

## Introduction

Mass spectrometry (MS) was first developed as a tool for measuring the masses and abundances of naturally occurring isotopes of the elements. MS has been used to elucidate the geological, marine, or atmospheric point of origin for molecules based on small differences in the abundance of stable isotopes including ^13^C, ^15^N, and ^2^H.^1,2^ This ratio can be used to determine the diet and migration patterns of live animals and archeological specimens.^3,4^ Diet-influenced isotope ratios can also be used for food authentication.^5^ MS is also commonly used for radio-carbon dating of ^14^C for pre-1945 archeological samples^6^ and bomb pulse dating for samples post-1945.^7^ All of the aforementioned analyses are typically performed on specialized magnetic sector isotope ratio mass spectrometers (IRMSs), where the sample is pyrolyzed prior to MS analysis and then the isotope ratios are derived from the ratios of small molecules like H_2_, N_2_, CO_2_, and NO, among others. This method is highly sensitive, but the pyrolysis blends all of the isotopic contributions into a single pool to give a global average.

MS also plays a critical role in biochemical analysis using isotopes. Enriching a sample with stable isotopes can enhance the MS analysis. Isotopic labeling can introduce predictable mass shifts in the spectrum, which can be used for metabolite identification,^8^ protein quantification,^9^ and to measure rates of metabolism and lifetimes of molecules within organisms.^10^ Even without enrichment, the isotopic abundance of C, H, N, O, and S imparts a relative isotopic mass defect onto the first isotope peak, so its accurate mass can be used as a constraint for formula assignment.^11^

For other analyses, MS can play a supporting role, such as isotopic labeling of intact proteins for multidimensional NMR experiments. In these experiments, ^13^C and ^15^N are NMR-active, while their lighter counterparts are not, yet their abundances are only 1.1% and 0.4%, respectively.^12^ So, to obtain sufficient signal-to-noise (SN) ratio and resolution, it is desirable to enrich the protein with heavy isotopes. For overexpression in bacterial systems, including *Escherichia coli*, this process is simple due to robust growth in a simple, chemically defined medium containing [^13^C]-glucose and [^15^N]-ammonium chloride as the sole sources of carbon and nitrogen.^13^ However, to determine the structure of glycoproteins or proteins from eukaryotes, the expression must be performed in a higher organism with the necessary cellular machinery for glycosylation and protein folding.^13,14^ Eukaryotes have more complicated media requirements, and preparing fully labeled media for eukaryotes is expensive and time-consuming.^14−17^ Achieving a high incorporation of isotopic labels is critical, particularly for heteronuclear NMR experiments, as the efficiency of the experiment (η) scales as a product of the percent incorporation of the two isotopes, shown by the equation η = (% ^13^C) × (% ^15^N). If we also consider that the signal-to-noise (SN) scales as SN = η√*N*, where *N* is the number of scans, a 3% decrease in incorporation for both elements requires 13% more scans to achieve the same SN. Larger decreases in label incorporation can have compounding effects on the signal quality for heteronuclear NMR spectra.

Herein we report a strategy for determining isotopic incorporation for uniformly labeled proteins with both ^15^N and ^13^C at the peptide or intact protein level based on the observation of their isotope patterns. Our strategy involves simulating enriched isotope patterns over a range of ^15^N and ^13^C incorporation levels and fitting them to MS data, then taking the best-fitting isotope pattern as the appropriate incorporation level. There are currently many isotope pattern simulators available to use, including several free browser-based software like MS-Isotope (Protein Prospector) and enviPat, among others; however, none of these programs allow the modification of the isotopic abundances from natural abundance, and their graphical user interfaces (GUIs) are not conducive to rapidly generating and testing hundreds or thousands of distributions. IsoPro 3.0 by Mike Senko^18,19^ was used for our previous work with Shenoy et al.^20^ because it does support the modification of isotopic abundances, but was not efficient for generating and comparing simulations for a range of isotopic enrichment levels. For a search and match approach to finding the best fit of isotope patterns, it is desirable to have an isotope pattern generator that can be called as a subroutine from within the grid searching program and that allows isotope enrichment to be varied on demand.

Rockwood has reported several algorithms for calculating isotopic envelopes.^21−24^ His FFT-based algorithm^22^ is currently implemented in the MATLAB function isotopicdist, and the higher efficiency algorithm by Rockwood and Haimi^21^ is currently implemented as an interactive command line C++ script, as well as a javascript version, js-emass.^25^ Additionally, there are python-based isotope simulators pyISOPACh and PythoMS^26^ as well as mfapy,^27^ which is designed for ^13^C metabolic flux experiments. IsoSpec is another popular simulator with bindings to Python (IsoSpecPy), R (IsoSpecR), and C. There are also more recent offerings like ElemCor,^28^ which specialized in the simulation of isotopically enriched metabolites; NEUTRONSTAR, which is optimized for the efficient calculation of large molecules;^29^ and DEUTERIUM, which extends Rockwood’s FFT algorithm into a 2D FFT algorithm for enhanced detail.^30^

Here we present a straightforward approach for fitting simulated isotope patterns to experimental data using a modification to the MATLAB isotope simulator, isotopicdist. The new function, isotopicdist2, retains all of the properties of the original function but adds pairwise inputs that permit the user to modify the isotopic abundances of ^13^C and ^15^N on the fly. This approach allows scripts to call the simulator, fit isotope patterns and accurate masses, and assign enrichment levels. Issues with long runtimes and poor mass accuracy can plague simulations of high enrichment levels. To address these problems, we have investigated the substitution of isotopicdist by js-emass in the fitting algorithm. While js-emass cannot simulate the isotopic fine structure, it accurately determines the centroid of each isotope peak, allows isotopic abundances to be changed on the fly, and works with greater speed and mass accuracy than isotopicdist. With this approach, one can assign simultaneously [^15^N,^13^C] enrichment proportions in proteins and in their tryptic peptides. This has been used to optimize the enrichment protocol for a glycoprotein expressed in *Saccharomyces cerevisiae*.

## Experimental Section

### [^15^N,^13^C]-Ubiquitin Expression

Uniformly [^15^N,^13^C]-labeled ubiquitin was expressed in *E. coli* BL21(DE3) cells using M9 minimal medium containing 2 g/L [^13^C_6_]-glucose (Cambridge Isotope Laboratories, +98%) and 1 g/L ^15^NH_4_Cl (Cambridge Isotope Laboratories, 99%) as the sole carbon and nitrogen sources. The medium also contained 2 mM MgSO_4_, 2.4 mg/mL thiamine, and 100 μM CaCl_2_. An *E. coli* glycerol stock transformed with the pET:Ubiquitin expression vector (a gift from Vincenzo Venditti, Iowa State University) was streaked out on an LB agar plate containing 100 μg/mL ampicillin. Colonies were picked and used to inoculate a 5 mL overnight culture with 100 μg/mL ampicillin, which was incubated at 37 °C and 190 rpm overnight. The overnight culture was centrifuged at 6000 g for 10 min, and the supernatant was removed. The cell pellets were resuspended in 1 L of minimal medium with ampicillin and incubated at 37 °C until the cultures reached an OD of 0.5. Protein expression was then induced with 1 mM isopropyl β-d-1-thiogalactopyranoside (IPTG), and the samples were incubated overnight at 18 °C and 190 rpm. Cells were harvested by centrifugation at 4500 g for 15 min, and the cell pellets were resuspended in lysis buffer (50 mM MOPS, 100 mM NaCl, pH 7.2). The cells were lysed by a French press between 1000 and 1500 psi. Cell debris was removed by centrifuging at 20000 g, and the supernatant was heated at 90 °C for 5 min using a water bath to precipitate other proteins. The precipitated proteins were pelleted by centrifugation at 20 000 g for 45 min at 4 °C. The supernatant was collected and concentrated using 3 kDa MWCO filters (Millipore Amicon Ultra-15). The concentrated supernatant was loaded into a 10 mL superloop on an Äkta Go system (Cytiva) and ran across a HiLoad 26/60 Superdex 75pg size exclusion column (Cytiva) using a method designed in Unicorn 7 (Cytiva). The [^15^N,^13^C]-ubiquitin was further purified using a Superdex 75 size exclusion column (Cytiva) pre-equilibrated with 1× PBS.

### S75 Protocol

For the Superdex 75pg purification, a 25 mM MOPS (Sigma-Aldrich) and 100 mM NaCl (Sigma-Aldrich) buffer (pH 7.4) filtered using a 0.2 μm filter (VWR) was used as the running buffer. The column was first equilibrated at a flow rate of 0.5 mL/min with 0.01 CV of running buffer, with the UV detector being zeroed at the end of equilibration. The sample was then injected onto the column from the superloop using 11 mL of running buffer at a flow rate of 0.5 mL/min. The protein was then eluted with 0.8 CV of running buffer at a flow rate of 0.5 mL/min, with 5 mL fractionation delayed until after 0.35 CV had passed across the column and ending after the elution phase. The column was then washed with 0.45 CV of running buffer at a flow rate of 0.5 mL/min. Fractions were then run on an SDS-PAGE gel to determine which fractions contained ubiquitin.

### [^15^N]- and [^15^N,^13^C]-IgG1 Fc Expression in Yeast

All expression protocols for the [^15^N] and [^15^N,^13^C]-IgG1 Fc were reported as previously described by Shenoy et al. and Davis et al.^20,31^ The improved IgG1 Fc expression was also reported by Davis et al.^31^ In order to increase ^13^C and ^15^N incorporation into the protein, a homemade heavy-isotope-labeled yeast extract was generated prior to protein expression as described.

### Trypsin Digest and MALDI Sample Preparation

Approximately 10 μg of each protein was digested with trypsin (Promega, Trypsin Gold, mass spectrometry grade) overnight at 37 °C. Following the digestion, the peptides were desalted using a C_18_ Zip-Tips. Trifuoroacetic acid (TFA; Fisher 99.9%) was added to each sample for a final concentration of 1% (v/v). The peptides were bound to the C_18_ resin and washed with a 0.1% TFA solution in water. The peptides were eluted into 10 μL of 50:50 acetonitrile/water (ACN, HPLC grade; H_2_O, HPLC grade, Sigma-Aldrich). MALDI spots were prepared by spotting 0.5 μL of protein solution onto an MTP AnchorChip 384 MALDI plate (Bruker) and allowing the solution to dry fully. Then, 0.5 μL of 2,5-dihydroxybenzoic acid (DHB; Alfa Aesar 99%; 15 mg/mL in 50:50:0.1 MeOH/H_2_O/formic acid (FA; Sigma-Aldrich, 98—100% LC–MS grade)) was spotted on top of each peptide spot and allowed to dry fully. If good DHB crystals did not form, then a series of dilutions were performed on the MALDI plate to reduce the peptide concentration.

### Intact Protein Sample Preparation for Mass Spectrometry

Intact natural abundance ubiquitin (bovine, P0CG53) was obtained as a lyophilized solid. A stock solution was prepared at 1 mg/mL in H_2_O, which was then diluted 1:10 in 50:50:0.1 MeOH/H_2_O/FA prior to MS analysis. [^15^N,^13^C]-Ubiquitin (10 μL, 10 mg/mL in 20 mM NaHPO_4_) was desalted using a C_18_ ZipTip (Millipore, size P_10_) using the standard ZipTip protocol. The protein was then eluted into 10 μL of MeOH/H_2_O/FA (50:50:0.1) and loaded into a borosilicate glass nanoelectrospray emitter with an orifice size of approximately 1 μm.

Each Fc protein intact measurement solution was buffer exchanged into 50 mM ammonium acetate using 10 kDa MWCO filters (Thermo Pierce concentrator 10 k MWCO 0.5 mL). The proteins were subjected to up to ten rounds of centrifugation and buffer washing with 50 mM ammonium acetate until [Na^+^] or [K^+^] was reduced to below 1 μM. The proteins were then diluted to 5 μM in 200 mM ammonium acetate. Prior to collecting data, 10 μL of protein solution was loaded into a homemade borosilicate nanoelectrospray emitter with a tip ID of 1 μm.

### FT-ICR Mass Spectrometry

Mass spectra were collected on a Bruker SolariX XR 12 T Fourier Transform Ion Cyclotron Resonance (FTICR) mass spectrometer equipped with a dual ESI/MALDI ion source and a dynamically harmonized ParaCell. Mass calibration for protein digests was performed with cesium iodide (CsI) (Aldrich 99.9999%) between 500 and 3000 *m*/*z* using ESI. Protein digests were ionized using MALDI with a SmartBeam II laser. The number of points was set to 512 000, which gave a transient length of 0.70 s and a resolving power of 130 000 at 600 *m*/*z*. 48 scans were averaged per spectrum. MALDI spectra for the uniformly enriched Fc digests are reported as previously described by Davis et al.^31^ To assess reproducibility, five replicate spectra were collected on the same day from different MALDI spots using the same parameters listed above. To assess signal averaging effects, a serial mode acquisition was performed on a single MALDI spot with >64 scans. Then, individual spectra were exported that averaged 1, 2, 4, 8, 16, 32, and 64 scans, each of which was analyzed separately.

Intact ubiquitin spectra were collected using ESI. Mass calibration was performed using sodium trifluoroacetate (NaTFA, Alrich 99%) between 150 and 3000 *m*/*z* with an RMS error of 0.1 ppm. Spectra were collected with 2 M points, which gave a transient length of 1.12 s and a resolving power of 100 000 (857 *m*/*z*) (10+ charge state of ubiquitin). 48 scans were averaged per spectrum. Intact protein mass spectra were deconvoluted using MaxEnt (Bruker Compass Data Analysis 5.1) to create a neutral spectrum, with the resolution set to 100 000. To assess reproducibility, five replicate ESI spectra were collected in the same data using the parameters listed above ensuring <1 ppm mass accuracy. Each spectrum was deconvoluted with MaxEnt as described above.

### Isotope Distribution Simulations

Isotope patterns were simulated in MATLAB’s built in isotope simulator “isotopicdist”, which uses the isotope pattern calculation algorithm reported by Rockwood et al.^23^ and was modified to allow for the isotopic abundances of ^13^C and ^15^N to be procedurally modified. Rockwood’s algorithm relies on the convolution theorem, which states that convolution in one domain corresponds to pointwise multiplication in the transformed domain. The calculation of a heterodyned isotope distribution, *F*(*m* – *m*_0_), is shown by
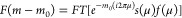
1where *m* is the mass of the molecule and *m*_0_ is the heterodyne mass, which is chosen to shift the distribution close to 0 Da in order to reduce the number of points required to satisfy the Nyquist sampling theorem. *s*(μ) is a function used to include peak shape in the convolution, the inverse Fourier transform of a peak shape function in the mass domain (*m*), which is typically Gaussian, though other peak shapes may be used.
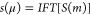
2

The peak shape function has the added advantage of variable peak width, so simulations can be generated at any desired fwhm, all the way down to simulating the isotopic fine structure. Finally *f*(μ) includes information about the elements present in the molecule and their isotopic abundances, as shown by
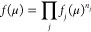
3where *f*_*j*_ is defined by
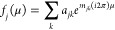
4where *a*_*jk*_ is the *k*th isotope abundance of the *j*th element in the molecular formula, and *m*_*jk*_ is the mass of the *k*th isotope of the *j*th element in the formula. However, for discrete FFT calculations, *f*_*j*_ can be defined as
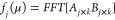
5where *A* is a *j* by *k* matrix containing the masses of all *j* elements in the formula as well as their heavy isotope masses and *B* is a matrix containing the relative isotopic abundances of each element and their respective isotopes. For the proteins simulated in this paper, typically only C, H, N, O, and S are considered, so
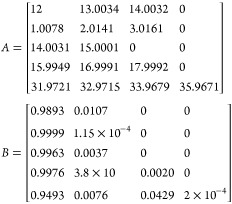
6

Each of these values was rounded to four decimal places for visibility but was known to 7–10 decimal places in the script. The MATLAB function isotopicdist can accept either a protein sequence or an elemental formula to begin a calculation, but by default it cannot simulate isotopically enriched molecules without manually changing the values within *A* or *B*. In order to test and fit multiple distributions rapidly, new pairwise inputs were added to the isotopicdist function to allow the isotopic abundances for ^13^C and ^15^N in *B* to be modified with every call to the function, so long as the values are between 0–100%. For example, the input isotopicdist(“PEPTIDE”, “C13abund”, 99) changes the ^12^C abundance to 1% and the ^13^C isotopic abundance to 99%, which are later converted to 0.01 and 0.99, respectively.
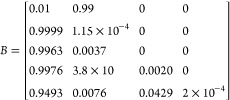
7

**Figure 1:**
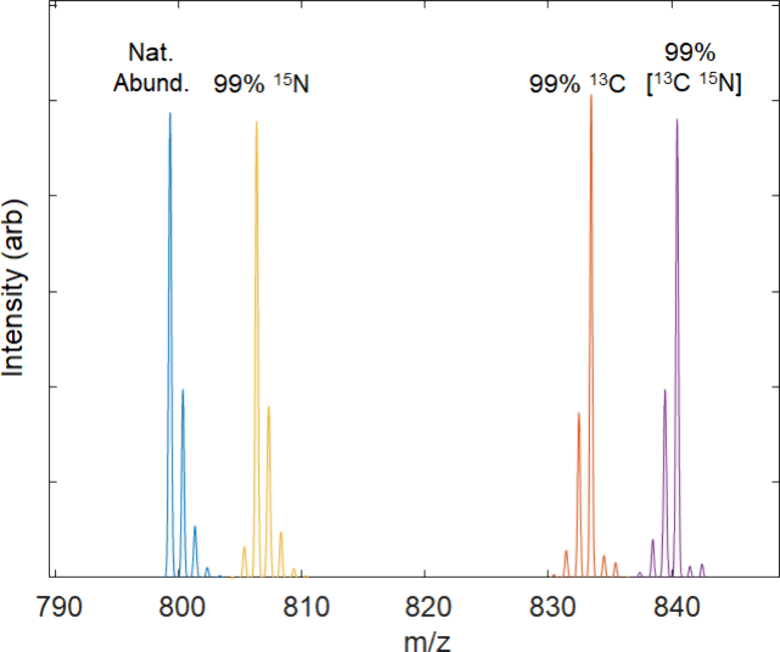
Simulated isotope distributions for the peptide “PEPTIDE” at natural abundance (blue) and uniformly enriched with 99% ^15^N (yellow), 99% ^13^C (orange), and 99% [^15^N,^13^C] (purple).

This will, in effect, generate an isotope distribution that has its ^13^C content uniformly enriched to 99%. Similarly, the ^15^N content can be set to 99% with the call isotopicdist(“PEPTIDE”, “N15abund”, 99). Also, both elements can be enriched simultaneously by submitting both inputs, e.g. isotopicdist(“PEPTIDE”, “N15abund”, 99, “C13abund”, 99). Figure 1 shows the resulting peptide simulations from these calls.

It should also be noted that other than these changes, isotopicdist has remained largely unmodified. There are several other calculations detailed by Rockwood to optimize the calculation for speed and conservation of memory. The fft window size calculation, number of points in the fft, and the monoisotopic mass, among others, remained largely unmodified. However, these parameters have all been optimized for natural abundance simulations, not enriched simulations, so the calculation time can be suboptimal for larger species (>10 s per simulation) and has significant mass error (>5 ppm for peptides, >15 ppm for intact proteins) when simulating isotope patterns close to 100% enrichment.

For this reason, an alternate isotope simulator, js-emass, was integrated into our software, which is a JavaScript implementation of Rockwood and Haimi’s emass.^21,25^ emass uses an different algorithm than isotopicdist; it calculates the centroid peak positions for each isotope peak by successively combining superatoms together until the desired elemental formula is reached, and it prunes low abundance terms from the calculation to save time. js-emass provides a set of JavaScript functions based on the emass algorithm, which also allows the user to procedurally modify the abundance of any isotope. This was also implemented in DEUTERATER, which is used for ^2^H labeling applications.^32^ However, most of our fitting and analysis software was already developed in MATLAB (described below), so a MATLAB function was developed to control js-emass from the command line. This function, named emass for convenience, accepts a peptide sequence or an elemental formula and has optional pairwise inputs “C13abund” and “N15abund” to modify the abundances of ^13^C and ^15^N, respectively. Despite needing to bridge two programming languages, js-emass simulates isotope patterns for enriched peptides and proteins considerably faster than MATLAB’s isotopicdist.

### Fitting Isotope Patterns to Experimental Data

In order to experimentally determine ^15^N or ^13^C incorporation levels, we developed a script that uses a test-and-fit strategy. The script generates enriched isotope distributions across a user-specified range of ^15^N or ^13^C. If both elements are treated as unknowns, the abundances of ^15^N and ^13^C are changed in nested loops, and the simulations are tested against the data using a grid search. Each simulation is fit against the data using either RMSE (root-mean-square error) or *Χ*^2^ depending on the user’s preference.
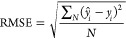
8
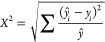
9

**Figure 2:**
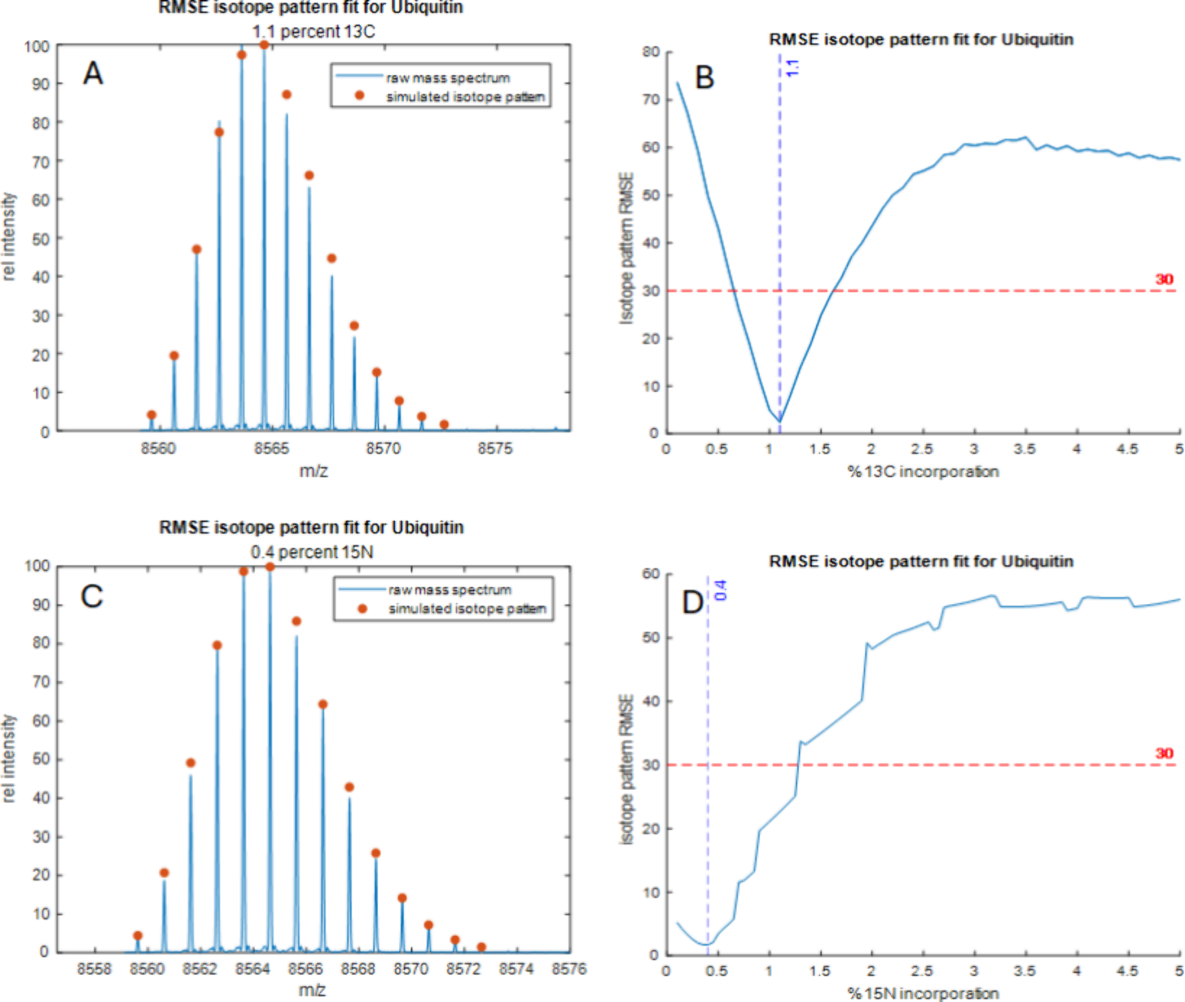
(A) Deconvoluted mass spectrum for ubiquitin overlaid with a simulated isotope distribution at 1.1% ^13^C. (B) RMSE fits for simulated ubiquitin as a function of ^13^C. (C) Deconvoluted mass spectrum for ubiquitin overlaid with a simulated isotope distribution at 0.4% ^13^C. (D) RMSE fits for simulated ubiquitin isotope distributions as a function of ^15^N.

**Figure 3:**
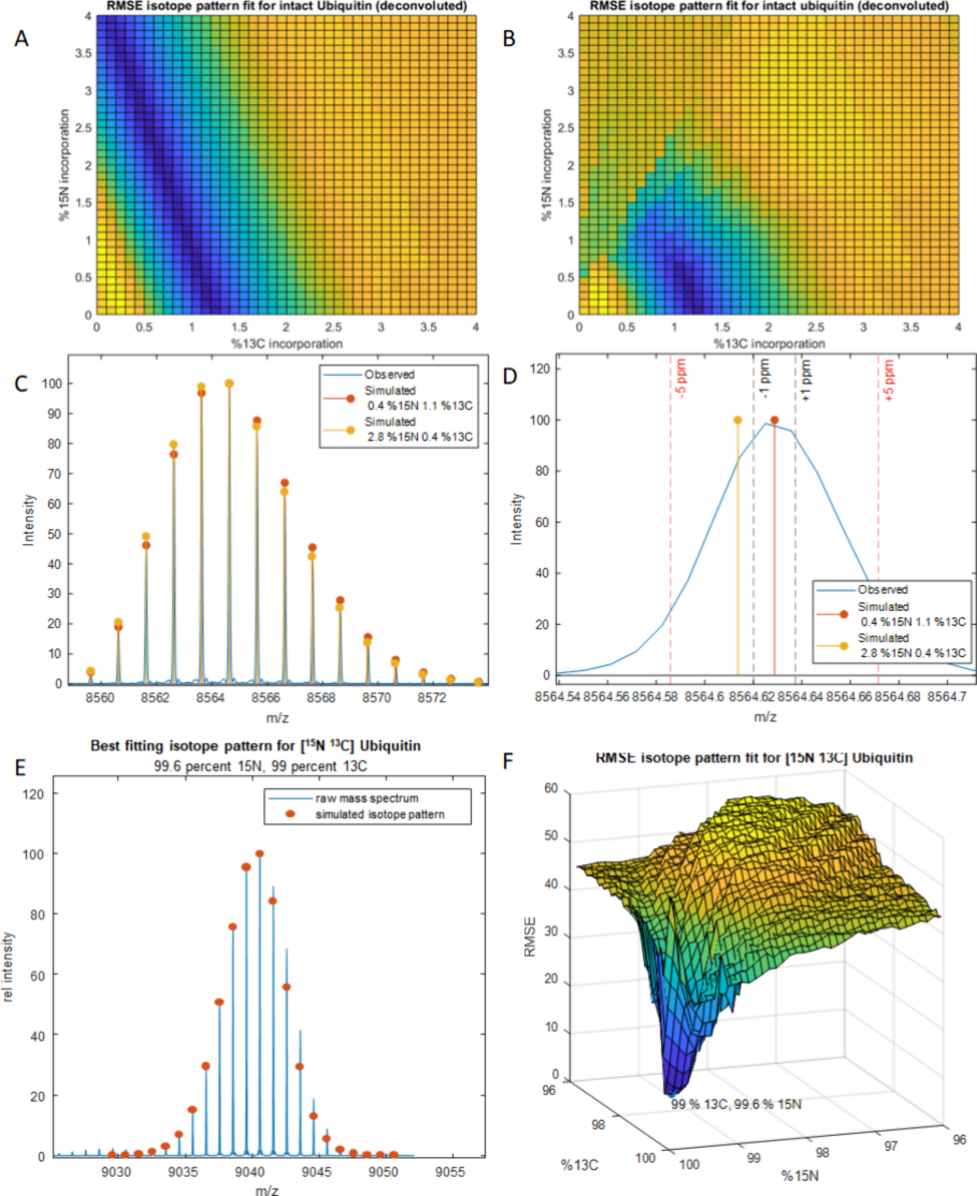
(A) RMSE surface plot for isotope distribution fits to intact ubiquitin using a 5 ppm error cutoff. (B) RMSE surface plot for isotope distribution fits to intact ubiquitin using a 1 ppm error cutoff. (C) Deconvoluted mass spectrum for intact ubiquitin overlaid with two simulated distributions. The first distribution in orange has the correct isotopic contributions for N and C, while the distribution in yellow has incorrect isotopic contributions but a passing RMSE score. (D) Expansion of (C) on the most abundant isotope, which shows how the incorrect simulation (yellow) is shifted in mass by <1 ppm despite having the correct shape for the overall isotopic envelope. (E) Deconvoluted spectrum for uniformly [^15^N,^13^C]-enriched ubiquitin. (F) RMSE surface plot for [^15^N,^13^C]-ubiquitin simulations as a function of ^15^N and ^13^C.

Here *ŷ*_i_ is the intensity of the *N*th peak of the theoretical isotope distribution, *y*_i_ is the intensity of the corresponding isotope peak in the experimental data, and *N* is the number of isotope peaks in the simulation. Additionally, there is a user defined ppm error cutoff which is used to define how far a peak in the data may be located from its simulated counterpart. If a simulated isotope peak does not fall within the range of any peaks in the data, *y*_i_ is set to 0, so the numerator is equal to *ŷ*^2^. If none of the simulated peaks line up with the data, they are all fit against *y*_i_ = 0, which usually results in an RMSE value of >45, while very well-fitting distributions have RMSE values <10. After all RMSE values have been calculated, an RMSE plot is generated (Figure 2a and d) which should contain a minimum with the correct isotope incorporation. If only one element is treated as an unknown, then the minimum is detected procedurally by inverting the RMSE plot and detecting the peak using a peak picking function in MATALB, “findpeaks”. This helps to ensure that the selected isotope incorporation is chosen from a well in the RMSE plot rather than the global minimum at the edge of the plot, especially if the global minimum has a lower RMSE than the correct answer. If two elements are treated as unknowns, a 2D RMSE surface is generated (Figure 3B), which should contain an RMSE well containing the correct answer. Procedural detection of the RMSE well has not yet been implemented, so instead the global minimum (or lowest RMSE value) is selected as the correct answer. In many cases this will coincide with the well in the plot, but it can cause false convergence at the edge of the plot, even when an obvious local minimum can be seen in the surface.

The script will accept a list of amino acid sequences or elemental formulas, which makes it useful for assessing incorporation levels across entire tryptic digests and allows it to analyze nonprotein molecules such as glycans. If a list of amino acid sequences is submitted, then they will be converted to neutral elemental formulas or singly charged [M + H]^+^ ions, depending on which is specified by the user, so only MALDI or deconvoluted spectra may be used.

## Results and Discussion

To assess the capability of this approach to accurately assign the isotope abundances for ^13^C and ^15^N, we first tested this on two simple fringe cases, ubiquitin containing the natural isotope abundance and ubiquitin that was +99% uniformly enriched with ^15^N and ^13^C. We recognize that these two cases in particular are trivial to measure but help to establish analytical considerations for this technique and aid in the interpretation of the RMSE plots presented later. The expected natural abundance of ^13^C is 1.1%, and the abundance of ^15^N is 0.37%.^11^ First, this strategy was applied to a deconvoluted electrospray mass spectrum of intact ubiquitin acquired at a resolving power of 100 000 (Figure 2a, Figure S2), which provides isotopic resolution but does not resolve the isotopic fine structure. If only one element is treated as an unknown, the assignment of its heavy isotope abundance is trivial. We were able to assign the ^13^C abundance at 1.1% by fixing the ^15^N abundance at 0.37% using 0.1% step sizes, as shown in Figure 2b. We also could derive a ^15^N abundance of 0.4% if the ^13^C abundance was set at 1.1% (Figure 2d). The minimum in the RMSE graph was determined by inverting the graph and applying a peak picking function to arrive at a single value, although it might be appropriate to fit a Gaussian curve to the RMSE plot and report a confidence interval; however, this has not yet been implemented in our software. The shape of the RMSE plot is not always Gaussian, so this strategy may not be feasible in all cases. However, for the intended application, the isotopic abundances of both C and N must be treated as unknowns. Figure 3a shows a root-mean-square error (RMSE) surface plot generated from a two-dimensional search using different parts per million error tolerances, where the blue regions correspond to low RMSE scores and the yellow areas are high scores. If a 5 ppm tolerance is used (Figure 3a), then many linearly related combinations of ^13^C and ^15^N will fit the isotope pattern with similar RMSE scores. Figure 3c shows an observed ubiquitin spectrum overlaid with two simulations with passing RMSE scores from Figure 3a, one with the correct isotope abundances and the other with a low ^13^C abundance (0.4%) and higher ^15^N abundance (2.8%). The two simulations are visually similar, and the simulation with the incorrect isotopic abundances actually produces a lower RMSE than the correct answer. When the error tolerance is reduced to 2 ppm (not shown), the region of well-fitting isotope patterns shrinks but still encompasses a wide range of abundances. Finally, when the error tolerance is reduced to 1 ppm (Figure 3b), a sharp global minimum can be located, which in this case was 1.1% ^13^C and 0.3% ^15^N, accurate within 0.1% of the actual abundances.

The reason the mass tolerance impacts the RMSE is that changes in C and N isotopic content will introduce small changes in the position of the centroid of an isotope peak due to changes in the underlying isotopic fine structure. At the resolution used for acquiring these data (*R* = 100 000), the isotopic fine structure is not resolved. Nevertheless, the peak shape and centroid is determined by the distribution of the underlying, but unresolved, fine structure peaks.^33^^13^C imparts a positive relative isotopic mass defect (+0.003354 Da), and ^15^N imparts a negative relative isotopic mass defect (−0.00296 Da). Because of this, distributions with incorrect ^13^C and ^15^N contents are discounted due to their erroneous mass values. Figure 3d shows the fifth isotope peak of ubiquitin from Figure 3d overlaid with two simulations. At this level, the mass defect from the increased ^15^N content is more apparent. At 5 ppm tolerance, the simulation at 2.8% ^15^N is still close enough to its counterpart in the data and will survive into the RMSE calculation, but at 1 ppm it is sufficiently far away to be excluded from the fit. This illustrates the need for 1 ppm or better mass accuracy when simultaneously varying the enrichment levels of two elements and comparing the calculated isotope patterns to experimental data.

The reproducibility of these findings was assessed by collecting five replicate spectra of intact ubiquitin, ensuring 1 ppm mass accuracy on the monoisotopic mass. Each of the five spectra was analyzed, treating both ^13^C and ^15^N as unknowns with a 1 ppm error tolerance. Four out of the five replicates converged on 0.2% ^15^N and 1.1% ^13^C, and the fifth converged at 0.6% ^15^N and 1% ^13^C. Including the single outlier, this gave average values of 0.28% ± 63.9% ^15^N (mean ± relative standard deviation) and 1.08% ± 4.1% ^13^C. Signal averaging effects were also assessed for the intact protein by averaging spectra with 1, 2, 4, 8, 16, 32, and 64 scans (Figure S3). With only 1–2 scans, the experimental data fit a simulation for ubiquitin with an RMSE of ∼5, which is an acceptable low value. Between 4 and 8 scans, the RMSE was reduced to ∼2 and plateaued in that region all the way to 64 scans. So, for an intact deconvoluted protein, a relatively low number of scans may be acceptable.

This strategy was also employed to measure the enrichment levels of ^15^N and ^13^C in uniformly doubly labeled ubiquitin at the intact protein level. This construct was expressed in *E. coli* using standard uniform labeling protocols with +98% [^13^C_6_]-glucose and 99% ^15^NH_4_Cl as the sole sources of carbon and nitrogen, respectively.^34^ The incorporation level is expected to approach 98–99% under these growth conditions. For this two-dimensional search, both elements were treated as unknowns. The search grid tested [^15^N,^13^C] values with 0.1% steps between 96% and 99.9%. Figure 3e shows a deconvoluted mass spectrum for the [^15^N,^13^C]-labeled ubiquitin. Here we switched to js-emass to simulate isotope distributions due to the previously mentioned issues with run time and mass accuracy that isotopicdist has when calculating simulations close to 100% enrichment. The enrichment increased the average MW of the protein to 9040.567 Da (neutral). Additionally, the fully enriched peak (378 ^13^C, 105 ^15^N) appears at 9043.5669 *m*/*z* (theoretical mass 9043.5651 Da, 0.2003 ppm error). Figure 3f shows the RMSE surface plot for enriched ubiquitin distributions between 96% and 99.9% [^15^N,^13^C] with 0.1% step sizes and a 1 ppm error cutoff. From this calculation, the global minimum was determined at 99.0% ^13^C and 99.6% ^15^N (Figure 3f). It is also clear that the incorporation level is not 100% because the isotope pattern still resembles a Gaussian distribution instead of a single ^13^C/^15^N saturated peak.

**Figure 4:**
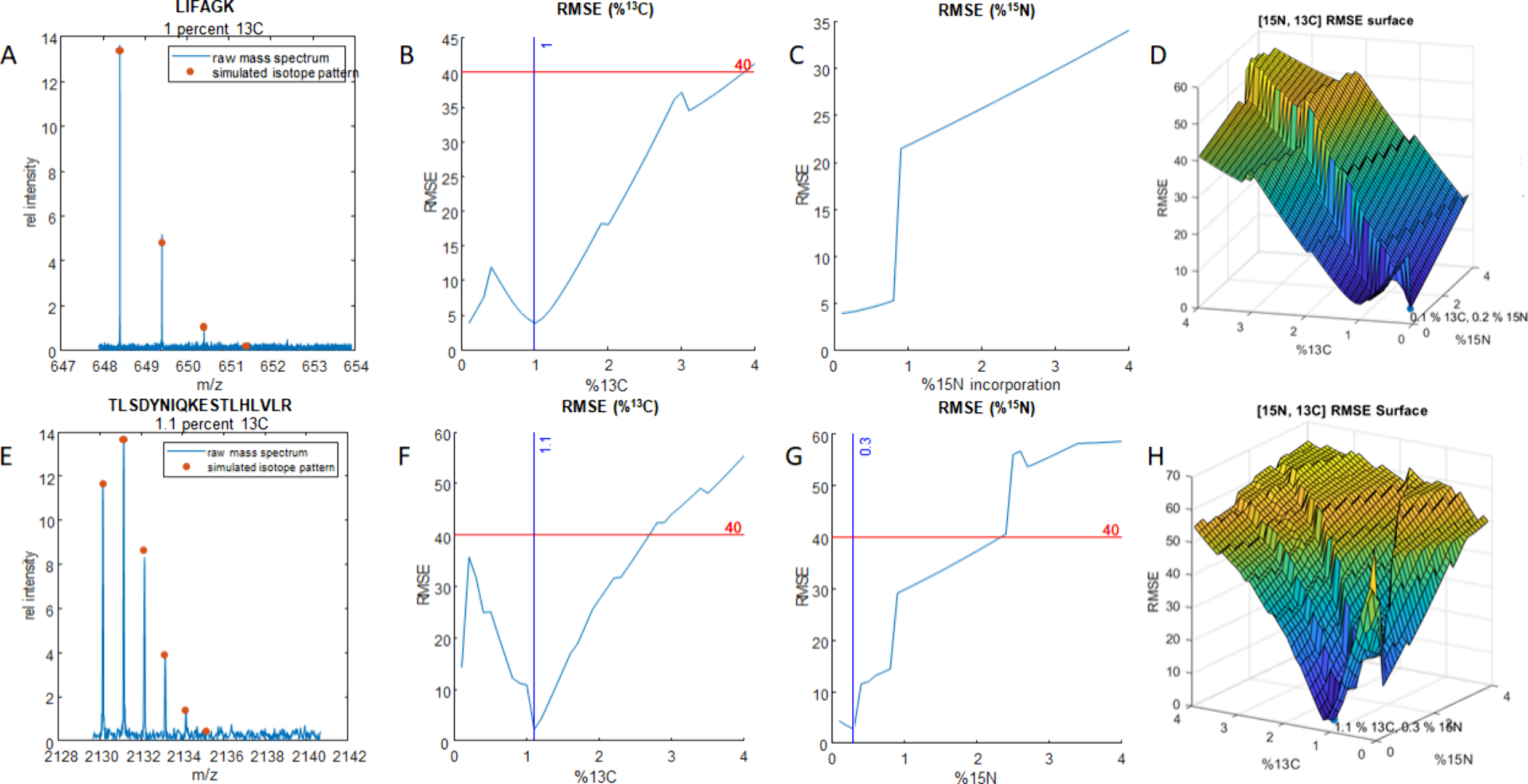
(A) MALDI mass spectrum for LIFAGK and a simulated isotope pattern with 1.1% ^13^C. (B) RMSE fits for simulated isotope patterns for LIFAGK as a function of ^13^C. (C) RMSE fits for simulated isotope patterns for LIFAGK as a function of ^15^N. (D) Two-dimensional RMSE plot for simulated isotope patterns for LIFAGK as a function of both ^15^N and ^13^C. (E) MALDI mass spectrum for TLSDYNIQKESTLHLVLR and a simulated isotope pattern with 1.1% ^13^C. (F) RMSE fits for simulated isotope patterns for TLSDYNIQKESTLHLVLR as a function of ^13^C. (G) RMSE fits for simulated isotope patterns for TLSDYNIQKESTLHLVLR as a function of ^15^N. (H) Two dimensional RMSE plot for simulated isotope patterns for TLSDYNIQKESTLHLVLR as a function of both ^15^N and ^13^C ranging from 0.1% to 4%.

Protein-level measurements of statistically accurate isotope distributions are challenging for high-molecular-weight proteins, particularly for heterogeneous samples, such as those with post-translational modifications like glycosylation. For this reason, we tested this analytical methodology at the peptide level. In general, calculations at the peptide level have larger relative errors than those for intact proteins because the much smaller numbers of carbon and nitrogen produce larger deviations from the statistical expectation, as has been previously addressed by Claesen et al.^35^ This can be addressed in part by selecting higher molecular weight peptides from the digest for this analysis. Low signal intensity also provides poorer agreement with statistical distributions for the similar reasons as smaller sample size, so more abundant peaks should be used as a selection criterion. To examine the peptide-level approach, the software was modified to take as input a list of peptide sequences and a MALDI spectrum, assessing each peptide composition sequentially. Advantages of performing this analysis at the peptide level are the accessibility to MS instruments with lower mass ranges and the ability to assess the statistics of fitting using multiple measurements. Figure 4 shows MALDI spectra for selected ubiquitin peptides for a nonenriched sample and their RMSE plots. Full MALDI spectra are shown in Figures S4 and S5. To examine the accuracy of the peptide-level approach, a single element was treated as an unknown. Eight peptides were automatically fit by fixing the ^15^N abundance to its native level (0.37%), and the average ^13^C enrichment level was determined to be 0.94 ± 0.12% ^13^C (Table S3). Measuring ^15^N incorporation was more challenging for peptides at natural abundance. The RMSE plots are more jagged (Figure S7), which caused issues with detecting the minimum procedurally, and many RMSE plots lacked a local minimum altogether. Typically, the number of nitrogens is only 20–30% that of carbons in any given peptide, and the heavy isotope contribution at natural abundance is only 0.4% per nitrogen atom; therefore, the influence of nitrogen on the isotope distribution is far less than that from carbon. Many ^15^N RMSE plots for this data set show a broad minimum covering a range of enrichment values, especially for lower MW peptides (Figure S7). However, there is an inflection point close to 0.4% ^15^N that could be found using manual inspection. In Figure 4c, the RMSE continues to decrease as the level of ^15^N enrichment approaches 0%. This is likely caused by our approach to calculating the fit, as we are matching a simulation to the peaks in the spectrum rather than fitting the spectrum to the simulation. Near 0% ^15^N, the simulation is essentially a monoisotopic peak with a very small first isotope, which, as it turns out, is very easy to fit against any other peak in the spectrum. Only 7 peptides contained a minimum that could be fit, which resulted in an average of 0.35% ± 0.20% ^15^N (Table S2). An alternative strategy for determining the % ^15^N incorporation at this level could be to use the isotopic mass defect of the first isotope, as described by Thurman et al.^33^

Next, simultaneous assignment of carbon and nitrogen enrichment was performed at the peptide level, again for a sample with a naturally occurring isotopic abundance. The same 8 peptides from trypsinolysis of ubiquitin were examined. Two peptides incorrectly converged on the edge of the RMSE surface rather than the local minimum at [ ∼1.1% ^13^C, ∼0.4% ^15^N]. The average isotopic incorporations including all 8 peptides was [0.76 ± 0.41% ^13^C, 0.25 ± 0.13% ^15^N], but excluding the two peptides that had a global minimum well below the other six, the isotopic abundances are measured to be [0.98 ± 0.53% ^13^C, 0.26 ± 0.15% ^15^N]. The process was more accurate for the larger peptides; as an example, the software found that TLSDYNIQKESTLHLVLR (Figure 4e and 4h) had 1.1% ^13^C and 0.3% ^15^N.

Reproducibility and signal averaging effects were also assessed for the peptides. On a separate day, five MALDI spectra of ubiquitin digests were collected, each from different MALDI spots. Peptides that appeared in all five spectra were analyzed by treating both ^15^N and ^13^C as unknowns. Seven peptides met this criteria, and their isotope incorporations are shown in Table 1. Measurement of ^13^C was highly reproducible across replicates, which generally had <10% RSD, but ^15^N had much greater uncertainty, likely for the same reasons discussed above.

**Table 1:** Isotopic Incorporation Determination for Ubiquitin Peptides with *n* = 5 Replicates

	LIFAGK	QLEDGR	MQIFVK	TLSDYNIQK	EGIPPDQQR	ESTLHLVLR	IQDKEGIPPDQQR
%^13^C	0.88	0.9	0.9	0.94	0.9	0.94	1.06
SD	0.084	0.000	0.000	0.055	0.000	0.055	0.055
RSD	9.5%	0.0%	0.0%	5.8%	0.0%	5.8%	5.2%
%^15^N	0.32	0.24	0.3	0.2	0.16	0.2	0.22
SD	0.179	0.089	0.000	0.122	0.055	0.100	0.164
RSD	55.9%	37.3%	0.0%	61.2%	34.2%	50.0%	74.7%

Signal averaging played a more significant role for peptides compared to the intact protein because the intensities of peptides can vary widely within a single spectrum. With only 1–2 scans, high-intensity peptides (>10%) had RMSE values between 2 and 10 and only saw marginal improvements in RMSE, if any, with signal averaging (Figure S6a). Lower intensity peptides (<10%) had RMSE values as high as 23 (Figure S6a) and generally saw a 1.3–2-fold improvement after 8 scans.

**Figure 5:**
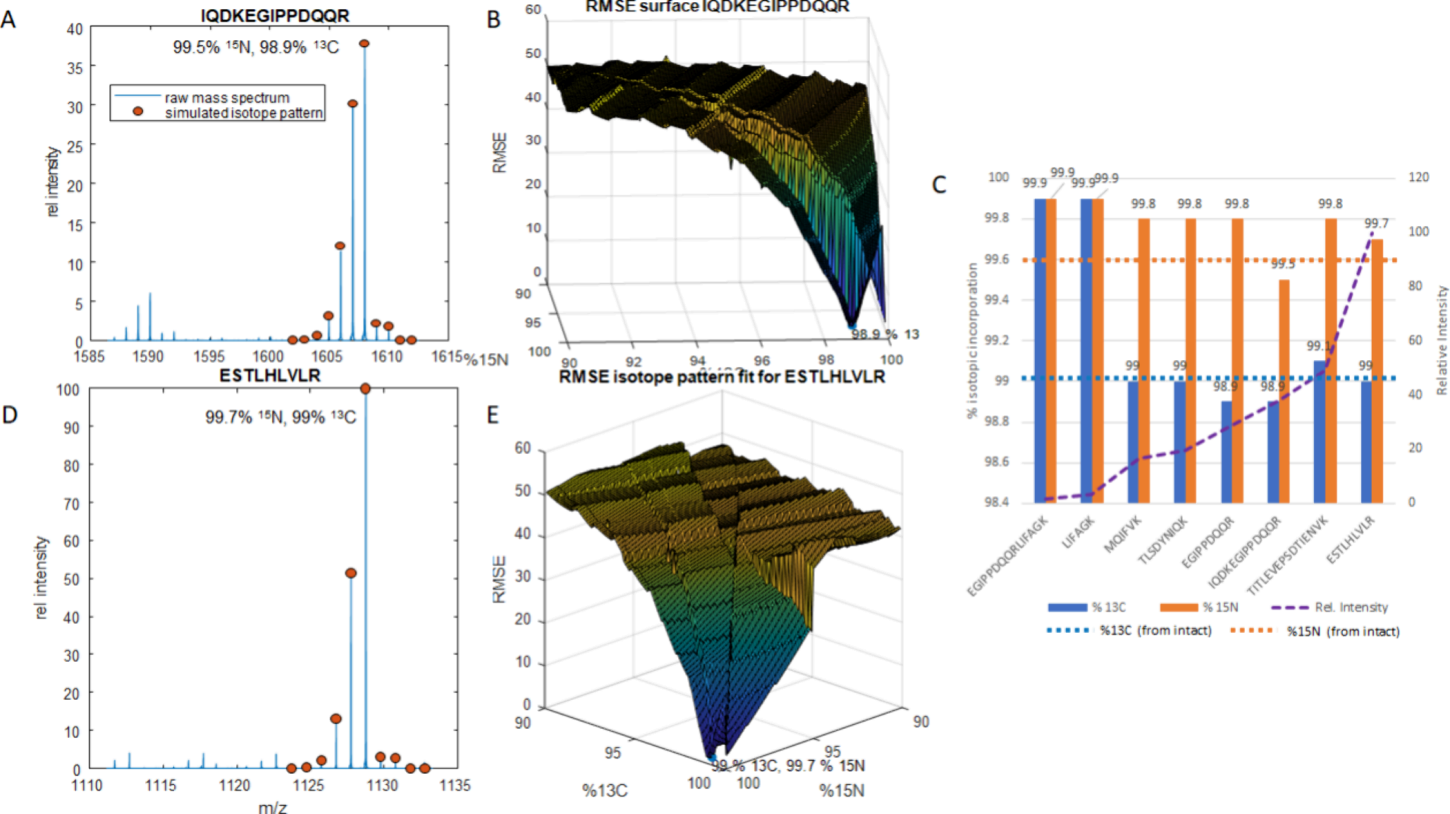
(A) MALDI spectrum of [^15^N,^13^C]-labeled IQDKEGIPPDQQR and a simulation of the same peptide with 99.5% N and 98.9% ^13^C. (B) RMSE fits for isotope pattern simulations of IDKIQDKEGIPPDQQR as a function of ^15^N and ^13^C. (C) ^15^N and ^13^C incorporations for each enriched ubiquitin peptide. Peptides are ordered by their relative intensity, as shown by the purple dashed line. (D) MALDI spectrum of [^15^N,^13^C]-labeled ESTLHLVLR and a simulation of the same peptide with 99.7% ^15^N and 99% ^13^C. (E) RMSE fits for isotope pattern simulations of ESTLHLVLR as a function of ^15^N and ^13^C abundance.

Fitting isotope patterns of peptides was more effective for isotopically enriched samples. For peptides derived from the uniformly labeled ubiquitin examined above at the protein level, it can be observed that most of the peptides converge at >98.9% ^15^N and >98.9% ^13^C. Most isotope distributions from this expression have the opposite shape from a natural abundance distribution (Figure 5a and d), where they monotonically increase up to the ^13^C/^15^N saturated isotope peak where all C and N atoms are replaced by their heavy counterparts. Then, two much smaller isotopologues follow the saturated peak, representing the contributions of ^2^H, ^17^O, ^18^O, and ^34^S if applicable. Were these peptides truly 100% incorporated, the isotope pattern would only consist of the C/N-saturated isotope peak followed by the small ^2^H and ^18^O isotope. Figure 5c shows the incorporation results that we received for each peptide, with each peptide sorted by its relative intensity from low to high. For most peptides, the best-fitting isotope patterns had ∼99.8% ^15^N and ∼99.0% ^13^C, which is consistent with the analysis of the intact protein above. Two peptides in this spectrum, LIFAGK and EGIPPDWWRLIFAGK, did not have a local minimum in their RMSE surface plot but rather converged at the edge of the plot at the global minimum instead. We believe that this is due to their low intensity in the mass spectrum. This is consistent with our earlier findings, as well as those by Claeson et al.,^35^ that low-intensity isotope distributions suffer from poor ion statistics and will deviate from statistical expectations. As such, an intensity cutoff was added to the software to automatically reject isotope patterns that fall below a user-defined threshold. Excluding the two lowest abundance peptides yields average values of 90.0 ± 0.1% ^13^C and 99.6 ± 0.4% ^15^N.

As expected, higher mass peptides tend to produce more accurate results, as mentioned above. They have more carbon and nitrogen atoms, more isotope peaks, and higher intensity isotope peaks, so the ^13^C and ^15^N have a more significant contribution to the isotope pattern compared to smaller peptides. This can be seen in the bar plot shown in Figure 5c, for which the measured values are shown for peptides ranging from 5 to 18 amino acids in length. For peptide-level isotope analysis, peak intensity and molecular weight are important factors in acquiring a statistically accurate isotope distribution.

### Analysis of [^15^N]- and [^15^N,^13^C]-IgG1 Fc Expressed with Yeast

**Figure 6:**
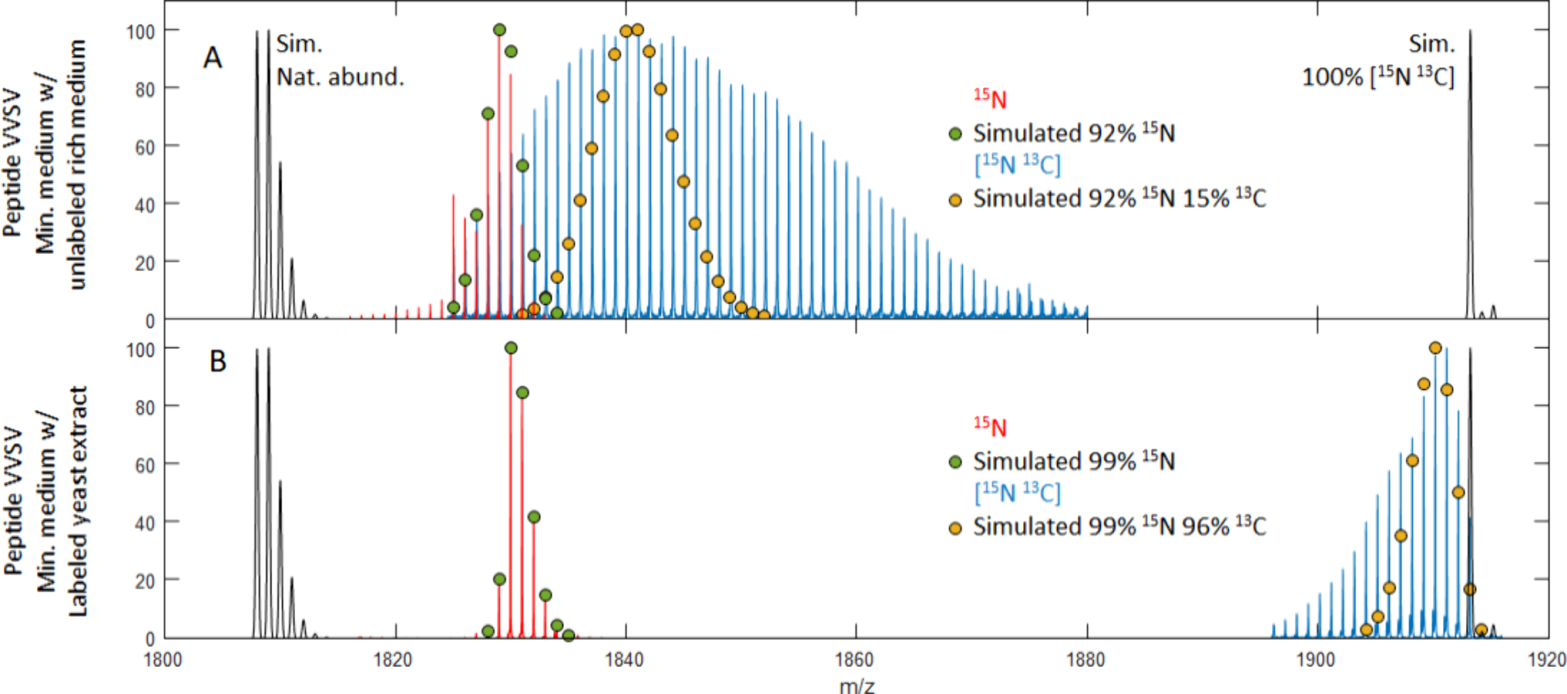
Mass spectra for the peptide VVSVLTVLHQDWLNGK that was uniformly enriched with ^15^N (red) and [^15^N,^13^C] (blue). Simulations for VVSVLTVLHQDWLNGK at natural abundance and 100% [^15^N,^13^C] are shown in black. (A) Original expression of Fc, which included 5% unlabeled yeast extract. (B) Improved expression with homemade heavy-isotope-labeled yeast extract.

This analysis methodology has been applied to an N-linked glycoprotein, human IgG1 Fc, expressed in *Saccharomyces cerevisiae*.^20,31^*S. cerevisiae* requires a more complex medium compared to *E. coli*, which contains additional nutrients in the medium in addition to [^13^C]-glucose and [^15^N]-ammonium salts. The initial test of this methodology used growth conditions with natural abundance nutrients and enriched glucose and ammonium salts for IgG1 Fc expression. Both [^15^N] and [^15^N,^13^C] enrichment were examined.^20,31^ We chose to analyze the tryptic peptides rather than the intact protein, as the glycoprotein glycan is highly heterogeneous and the individual glycoform peaks exhibit fairly low signal-to-noise ratios. The growth conditions resulted in a [^15^N] enrichment of 86 ± 7% measured at each peptide. The isotope pattern shown in red in Figure 6a is observed for tryptic peptide VVSVLTVLHQDWLNGK, with its corresponding simulation at 92% ^15^N shown in green. The simulation mostly agrees with the shape of the observed isotope distribution data, but there are some isotope peaks tailing to lower mass. We hypothesize that the deviation from the simulation is due to the presence of several overlapping levels of enrichment. This could be due to differential utilization rates for ^14^N compared to ^15^N. It is possible that ^14^N is being utilized more readily, which generates a small population of peptides of lower enrichment.

The expression of [^15^N,^13^C]-IgG1 Fc shows significant broadening in isotopic enrichment. Figure 6a (blue) shows the isotope distribution for the same peptide, which was typical for all the peptides in this data set (Figure S13). Each enriched peptide exhibited a much broader isotope pattern than predicted for uniform labeling. The data suggest that a range of enrichments are present and overlap each other. Our initial estimates of the isotopic incorporation were performed using isotopicdist,^31^ but its slow run times and issues with mass accuracy hampered our ability to treat both ^15^N and ^13^C as unknowns. To enable reasonable run times, we fixed the ^15^N enrichment at 92% (determined from the [^15^N]-Fc expression) and then solved for ^13^C. The broad isotope distribution made it difficult to assign a best fitting value, although several peptides did converge at the apex of the observed isotope distribution. This allowed us to determine that the average ^13^C incorporation was close to 30%, albeit with a large spread. The simulation in yellow in Figure 6a only has 15% ^13^C, but simulated patterns between 1.1% and 45% ^13^C (and 92% ^15^N) still fall within the observed isotopic envelope. Interestingly, we also obtained spectra for the oligomannose-type glycans from the same [^15^N,^13^C]-labeled IgG1 Fc expression, which were cleaved from the intact protein using CID (not shown).^31^ They had a much higher ^13^C incorporation of 91 ± 3% compared to the 30% in the peptide, likely due to the more efficient incorporation of [^13^C]-glucose into the glycan compared to the *de novo* synthesis of the amino acids from [^13^C]-glucose. These data illustrate an advantage of using this approach for measuring enrichment levels versus IRMS. The latter would give only a single value of enrichment for each element but no data regarding the distribution of enrichment levels.

Informed by these data, the expression protocol was modified with a supplement to the growth medium of a cell extract from yeast grown on [^15^N]- or [^15^N,^13^C]-enriched media. This significantly decreased the width of the isotope distribution and led to a much higher level of enrichment. Figure 6b shows the isotope distributions for the same peptide from the [^15^N] and [^15^N,^13^C] enrichment (red and blue respectively). The ^15^N peptides conformed closely to the simulations (green circles) and were enriched at a level of 98 ± 3% ^15^N. Also, the isotope distributions for the [^15^N,^13^C] double labeling were much narrower and had a much higher enrichment level, with an average value of 95 ± 4% ^13^C.^31^ The ^13^C enrichment still shows some skewing to lower mass, suggesting modest heterogeneity in the labeling, but clearly a much narrower distribution than in the first expression.

**Figure 7:**
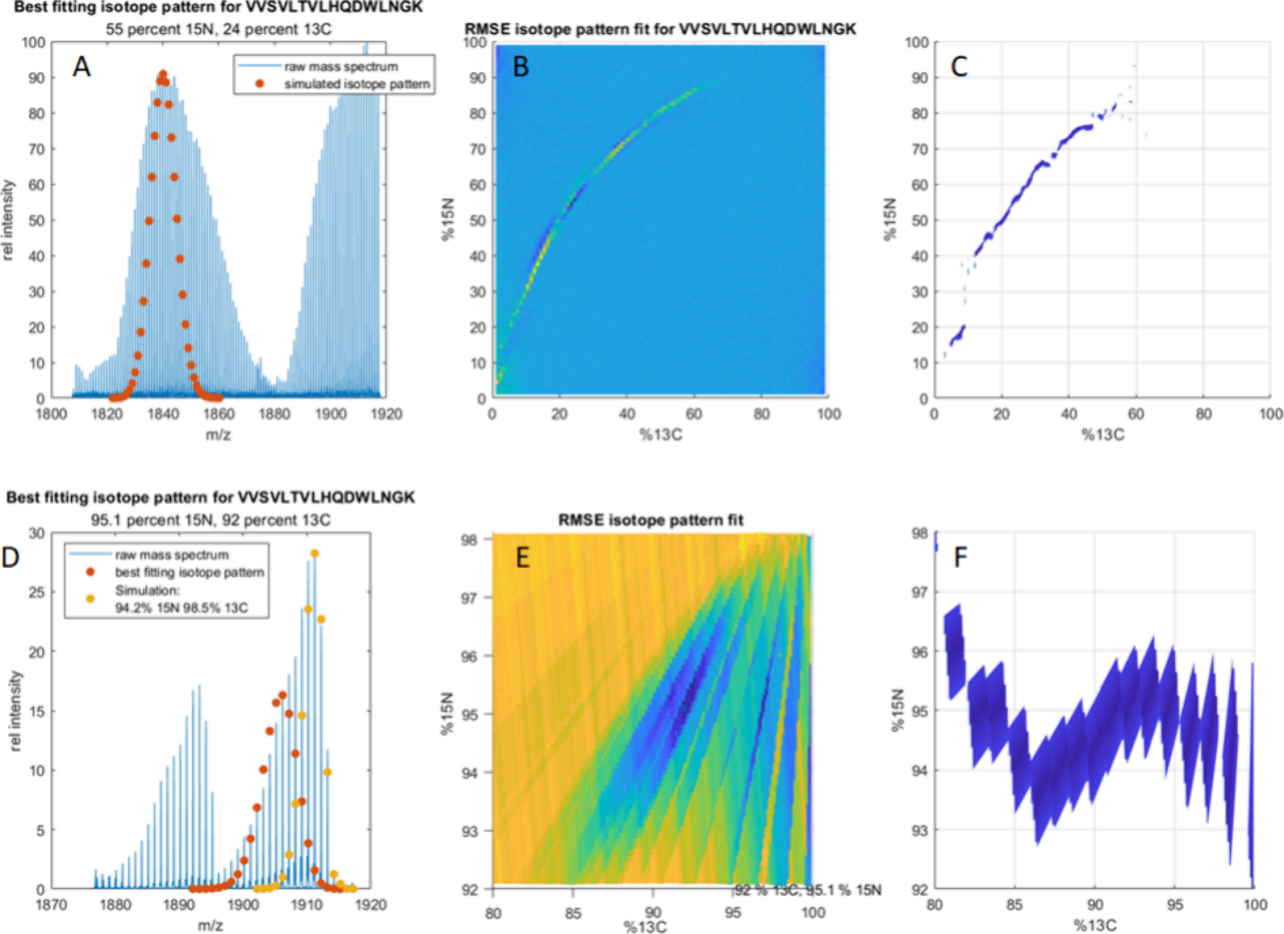
(A) MALDI spectrum of uniformly [^15^N,^13^C]-enriched VVSVLTVLHWDWLNGK with 5% unlabeled medium and a simulated isotope pattern (red) for the peptide with 55% ^15^N and 24% ^13^C. (B) RMSE surface plot for each simulated isotope pattern as a function of ^15^N and ^13^C abundance. (C) PPM errors between the most abundant peak in each simulation compared to their nearest neighbor as a function of ^15^N and ^13^C. All values above 1 ppm were removed for visibility. (D) MALDI spectrum of the improved uniformly [^15^N,^13^C]-enriched VVSVLTVLHWDWLNGK with heavy-isotope-labeled yeast extract, a simulated isotope pattern (red) for the peptide with 95% ^15^N and 92% ^13^C, and a second simulation (yellow) with 94.2% ^15^N and 98.5% ^13^C. (E) RMSE surface plot for each simulated isotope pattern as a function of ^15^N and ^13^C abundance. (F) PPM errors between the most abundant peak in each simulation and their nearest neighbor as a function of ^15^N and ^13^C. All values above 1 ppm were removed for visibility.

With the implementation of js-emass into the fitting program, we have been able to refine these measurements and perform searches with both ^15^N and ^13^C treated as unknowns. Figure 7 shows the results for 2D grid searches for [^15^N,^13^C]-VVSVLTVLHQDWLNGK from the original (Figure 7 a–c) and improved expression (Figure 7 d–f). The grid search in Figure 7 b and c was performed between 1% and 99% ^15^N and ^13^C. No single simulation conformed well to the data, but there were a range that had tolerable RMSE values (20–30) shown by the blue streak in Figure 7b. A similar range of ^15^N and ^13^C produced simulations whose most abundant isotope peak agreed closely (<0.5 ppm) with a neighboring peak in the data. To clarify, it is not that this technique is unable to converge on a single answer but that a range of isotope incorporations are contained within the observed isotope pattern. The range of incorporations for each element can be inferred from the best fitting regions of the RMSE and ppm surfaces, as the “best fitting” isotope pattern simulations are all contained within the observed isotope pattern. From the shapes of the well fitting areas in Figure 7b and c, it can be inferred that the ^13^C incorporation ranged from 15% to 60%, and the ^15^N incorporation ranged from 40% to 80% for this peptide.

A run was performed with the same parameters as above for the improved expression, but since the observed isotope patterns were much narrower compared to the initial construct, we performed a more refined search from 80% to 100% ^15^N/^13^C with 0.1% step sizes, 1 ppm error tolerance, and an RMSE cutoff of 40. As before, no single isotope pattern simulation conformed completely to the data, but we were able to produce a range of incorporations that might be contained within the observed isotope pattern. Figure 7d–f shows the results for the same peptide, VVSVLTVLHQDWLNGK, that was analyzed in Figure 7a–c. The observed isotope distribution for this peptide (Figure 7d) still does not conform to an ideal Gaussian isotope pattern, instead resembling a right-skewed χ^2^ distribution approaching 99%. Due to the shape of the distribution, we expect that single isotope incorporation will not produce a satisfying solution, but the range of incorporations that fall within this distribution will be smaller than that in Figure 7a–c. In fact, the best fitting isotope pattern (Figure 7a, orange) at 95.5% ^15^N and 92.3% ^13^C (RMSE = 21.5) falls within the observed distribution, but it appears that more intense isotopologues in the data approach 99% incorporation. Another simulation is shown in yellow in Figure 7d with 94.2% ^15^N and 98.5% ^13^C, which fills out the area of the observed distribution closer to 100% incorporation. These incorporations were chosen from a minimum in the parts per million surface in Figure 7f. The RMSE and parts per million surfaces in Figure 7e and f, respectively, show that a range of acceptable distributions fell between 94% and 96% ^15^N and between 89% and 99.9% ^13^C. In addition, we might also infer from Figure 7e and f that the dispersion of ^15^N incorporation is much lower than the dispersion of ^13^C incorporation.

To address the differences between the simulations and the observed isotope patterns in Figure 7, the isotope pattern simulators operate on an assumption that is fundamentally different from how biological systems process heavy-isotope-labeled media. The isotope pattern simulator generates an isotope pattern with a homogeneously mixed population of all isotopes and assumes that every amino acid in a given polypeptide has the same incorporation of ^13^C. For simulations with ^13^C or ^15^N incorporations between 5% and 95%, this will always yield an isotope pattern with a Gaussian distribution. However, in a biological setting, the ^13^C is not uniformly mixed with ^12^C, as they are provided to the organism in the form of [^13^C_6_]-glucose and [^12^C_6_]-glucose, each of which is used as a carbon source for de novo amino acid synthesis. The rates of de novo amino acid synthesis are subject to kinetic fractionation effects, so we anticipate ^12^C will be used at a marginally faster rate, roughly (12/13)^N^_,_ if it is present, where *N* is the number of enzymatic steps from [^12^C_6_]-glucose to a given amino acid. Also, as was the case in the first Fc expression in Figure 7a, some exogenous unlabeled amino acids were present in the medium that could be more rapidly utilized for protein transcription, in addition to other unlabeled carbon sources that could be utilized for de novo peptide synthesis. There is also evidence that, depending on the organism, ^13^C will not be uniformly incorporated into every amino acid if [^13^C_6_]-glucose is used in the presence of other unlabeled carbon sources.^36,37^ For all of these reasons, it cannot be assumed that the pools of ^12^C and ^13^C are uniformly mixed as viewed by the organism used as the expression system.

## Future Directions

One area that could be improved is simulating the isotope patterns that were observed in Figure 7a and d, as this could help to determine better statistics of the incorporation of each element. Two possible approaches could be utilized for this purpose, either generating linear combinations of many isotope pattern simulations and fitting them to the data or generating a more accurate simulation using a priori knowledge of the differential incorporation into each amino acid.

The linear combination approach could be performed after a grid search, as was performed in Figure 7. If we reference the ppm surface in Figure 7c, the blue region shows all ^15^N and ^13^C incorporations, which generated a simulation where the most intense peak agreed within 0.5 ppm of a peak in the experimental spectrum. Each ^15^N and ^13^C incorporation along the minima in Figure 7c could be used to generate new isotope pattern simulations, which could then be scaled individually and merged into a combined isotope pattern, *F*(*m*)_*c*_, shown by
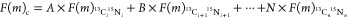
10where *A*, *B*, and *N* are simple scaling coefficients, and *F*(*m*)_^13^C_*i*_^15^N_*i*__ are each individual simulation with ^15^N and ^13^C incorporations determined from the minima of the ppm error surface. This would need to be incorporated into a fitting algorithm to determine the most appropriate scaling coefficient for each spectrum. We have implemented a basic version of this to illustrate the concept, but we have not yet designed a fitting algorithm to generate the linear combination. Figure S15a and b shows the isotope pattern for [^15^N,^13^C]-VVSVLTVLHQDWLNGK and its ppm error surface (duplicated from Figure 7a and c). As described above, the ^15^N and ^13^C values were taken from the minima of Figure S15b and used to generate the simulations in Figure S15c, which were scaled to match the height of their nearest neighbor peak in the data. As can be seen, the overlaid simulations in Figure S15c cover the same mass range as the observed pattern in Figure S15a, and the shape of the isotope pattern is also captured in the simulation. It is our hope that generating a linear combination of the individual simulations could produce a similar result and that the scaling coefficients could then be used to determine average and standard deviations for the incorporation of each element. This process was also repeated for the improved [^15^N,^13^C]-IgG1 Fc expression in Figure S15d–f. As can be seen in Figure S15f, the shape of the overlaid simulations reasonably captures the shape of the experimental data in Figure S15d.

The second method would require further modifications to the isotope pattern simulator to give different parts of the protein (i.e., amino acids or glycan vs backbone) independent isotope incorporations. This would hopefully be able to address the issue of simulating proteins with differential isotope incorporation but would require supplemental experiments on the protein similar to those performed by Hartel et al. and Willenborg et al.^36,37^ The protein would be hydrolyzed down to amino acids, which could then be profiled by using GC-MS or LC-MS in order to determine their ^13^C and ^15^N abundances. Once the incorporations of each amino acid are known, they can be used to inform a simulation. To give each amino acid an independent ^13^C incorporation, eqs 1 and 2 could be modified to split the population of carbon atoms into bins depending on the amino acid to which they belong to. For example, if we were to give proline and valine different ^13^C abundances compared to the rest of the protein, the vector that contains the elemental formula could be modified to
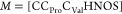
where C_Pro_ is the number of carbons that belong to proline, C_val_ is the number of carbons that belong to valine, and C = *C*_total_ – C_Pro_ – C_Val_. Then, to give proline 50% ^13^C, and valine 75% ^13^C, eq 6 would then be modified to
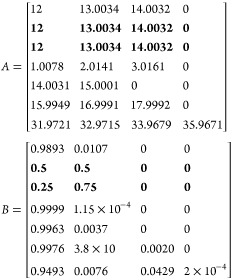
11

This could be extended to all 22 amino acids, which would require 22 carbon bins in the M vector and 22 rows for each carbon bin in the *A* and *B* matrices. This would hopefully generate an isotope pattern with a broader isotope pattern compared to a simulation where only the average incorporation was known, but it would not be able to account for kinetic effects that caused the experimental distribution to become broad. This method could also be used for glycopeptides and glycoproteins in order to separate the carbons into “protein” and “glycan” bins and give them separate ^13^C incorporations.

## Conclusions

The fitting of statistically derived isotope distributions with experimentally observed patterns provides an effective strategy for assigning the isotopic enrichment of ^15^N and ^13^C in doubly labeled proteins. This does not rely on resolving the isotopic fine structure but does require mass accuracy of 1 ppm or better. It can be employed with mass spectrometry data from any instrument that provides isotopic resolution and the aforementioned parts per million mass accuracy, including FTICR-MS, orbitrap mass spectrometers, and time-of-flight mass spectrometers. Compared to isotope ratio mass spectrometry, this approach can measure the distribution of enrichment values rather than just provide a global average. It also offers the advantage of being able to sample different parts of the protein (e.g., peptide/protein vs glycan in a glycoprotein) so their incorporation levels can be determined separately.^31^

A mass accuracy of 1 ppm or better is essential for determining the enrichment levels for two elements simultaneously. Isotopic enrichment changes the centroid of the isotope peaks (with the exception of the monoisotopic peak) due to changes in their underlying isotopic fine structures. A tight error tolerance helps to exclude incorrect enrichment assignments that might produce the correct abundance distribution but shift the exact masses of each isotope peak. A second important factor is to sample a large enough ion population to produce a statistically accurate distribution of isotopes. This can be achieved by selecting the most abundant components of a proteolytic digest and through a combination of signal averaging peak intensity.

## Data Availability

MATLAB code for determining isotopic abundances, the modified isotope distribution simulator, and the MATLAB code to control js-emass are available at https://github.com/erobertsFTMS/Uniform-Isotope-Labeling. JS-emass is available at https://github.com/emptyport/js-emass.

## Supporting Information Available

Additional mass spectra, replicate analysis and signal averaging analysis of intact ubiquitin, MS-Fit results for natural abundance ubiquitin peptides, ^15^N and ^13^C incorporations, RMSE plots, 2D RMSE surface plots, replicate isotope incorporation analysis of ubiquitin peptides, experimental isotope patterns of ubiquitin peptides and simulated isotope patterns with experimentally determined ^13^C and ^15^N, 2D ppm error plots for [^15^N,^13^C]-IgG Fc peptides, and overlay of many simulations for [^15^N,^13^C]-IgG Fc across a range of incorporations (PDF)
