# Supplementary material for: Automated Assignment of 15N And 13C Enrichment Levels in Doubly-Labeled Proteins
Source: J Am Soc Mass Spectrom. 2024 Aug 30;35(10):2344–57. doi: 10.1021/jasms.4c00218 (PMC11450805; doi:10.1021/jasms.4c00218)
Supplement: Supplementary file 1 — js4c00218_si_001.pdf [file js4c00218_si_001.pdf]

SUPPORTING INFORMATION

Automated Assignment of ^{15}N And ^{13}C Enrichment Levels in Doubly-Labeled Proteins

Elijah T. Roberts,[1] Alexander R. Davis,[2] Jeremy T. Risher,[1] Adam W. Barb,[1,2,3] I. Jonathan Amster[1]*

[1] Department of Chemistry, University of Georgia, Athens, GA, USA 30602

2 Department of Biochemistry and Molecular Biology, University of Georgia, Athens GA 30602 USA

3 Complex Carbohydrate Research Center, University of Georgia, Athens, 30602

* **Contact information for corresponding author:**

Department of Chemistry

1040K iSTEM-1

302 East Campus Road

University of Georgia

Athens, GA 30602-1546

Phone: 706-542-2001

Email: jamster@uga.edu

Table of Contents

Page

Figure S1: ESI mass spectra of ubiquitin and [^{15}N ^{13}C] ubiquitin S-3

Figure S2: Deconvoluted mass spectra of ubiquitin and [^{15}N ^{13}C] ubiquitin S-4

Figure S3: Reproducibility of isotope incorporation measurements, intact ubiquitin S-5

Figure S4: MALDI mass spectra of ubiquitin and [^{15}N ^{13}C] ubiquitin tryptic peptides S-6

Figure S5: Expansion of MALDI mass spectrum from Figure S4 .... S-7

Figure S6: Effect on signal averaging on the RMSE score of peptide isotope patterns S-8

Figure S7: RMSE plots for ubiquitin peptides; Determination of %^{15}N S-9

Figure S8: RMSE plots for ubiquitin peptides: Determination of %^{13}C S-10

Figure S9: RMSE surfaces for ubiquitin peptides: Determination of %^{15}N and %^{13}C S-11

Figure S10: Ubiquitin peptide isotope patterns and best fitting simulations S-12

Figure S11: RMSE surfaces, [^{15}N ^{13}C] ubiquitin peptides: Determination of %^{15}N and %^{13}C S-13

Figure S12: [^{15}N ^{13}C] ubiquitin peptide isotope patterns and best fitting simulations S-14

Figure S13: Isotope patterns, RMSE and ppm error surfaces for [^{15}N ^{13}C] Fc peptides................. S-15

Figure S14: Isotope patterns, RMSE surfaces, and ppm error surfaces for optimized enrichment of [^{15}N ^{13}C] labeled Fc peptides S-16

Figure S15: [^{15}N ^{13}C] Fc peptides: combining many isotope pattern simulations S-17

Table S1: Isotopic abundance determination results: intact ubiquitin replicates S-18

Table S2: MS-Fit results for ubiquitin peptides S-18

Table S3: %^{15}N determination for ubiquitin peptides S-19

Table S4: %^{13}C determination for ubiquitin peptides S-20

Table S5: %^{13}C and %^{15}N determination for ubiquitin peptides S-21

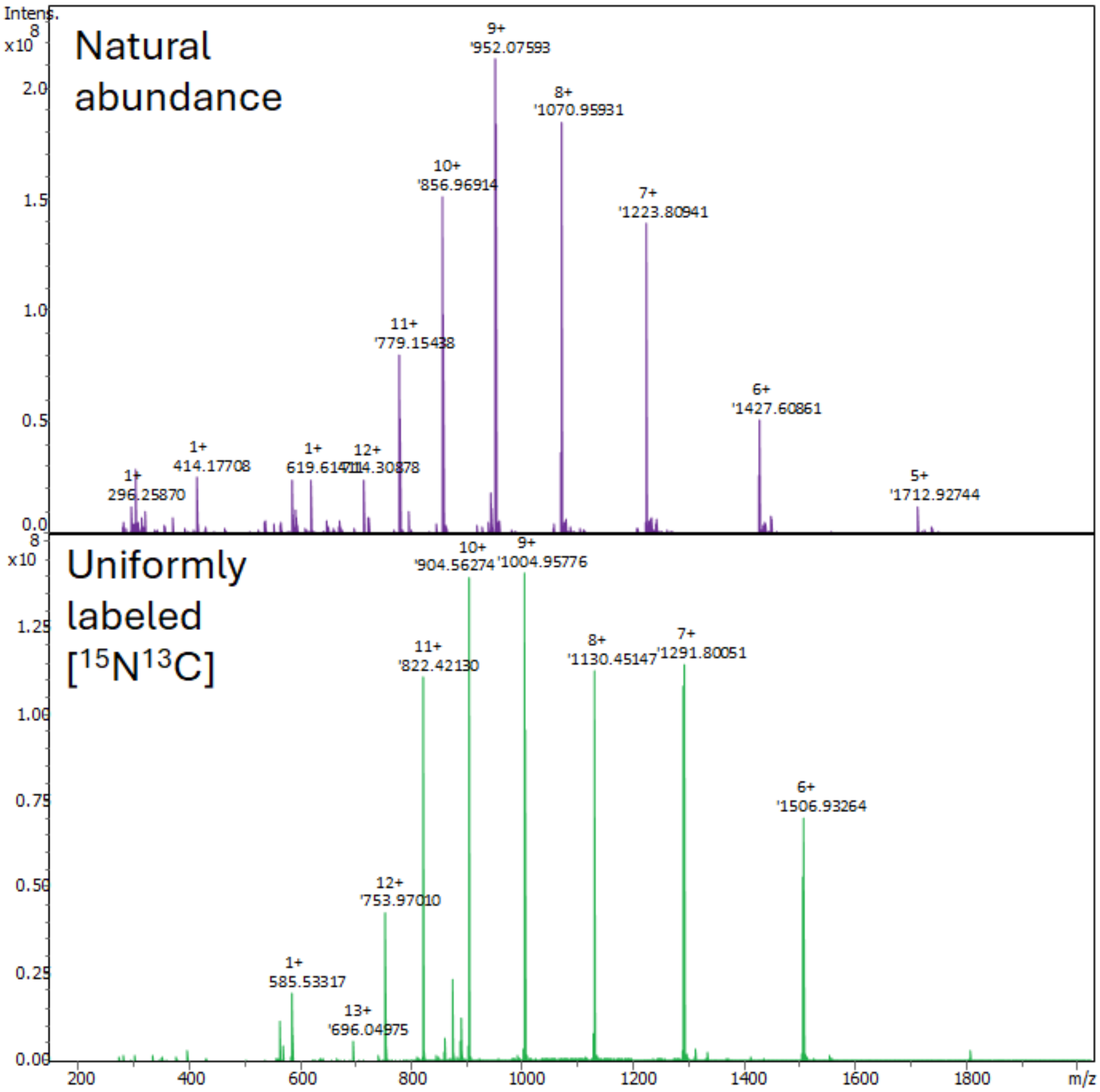

Figure S1: mass spectrum of intact ubiquitin (top) and mass spectrum of intact uniformly [^{15}N ^{13}C] labeled ubiquitin (bottom).

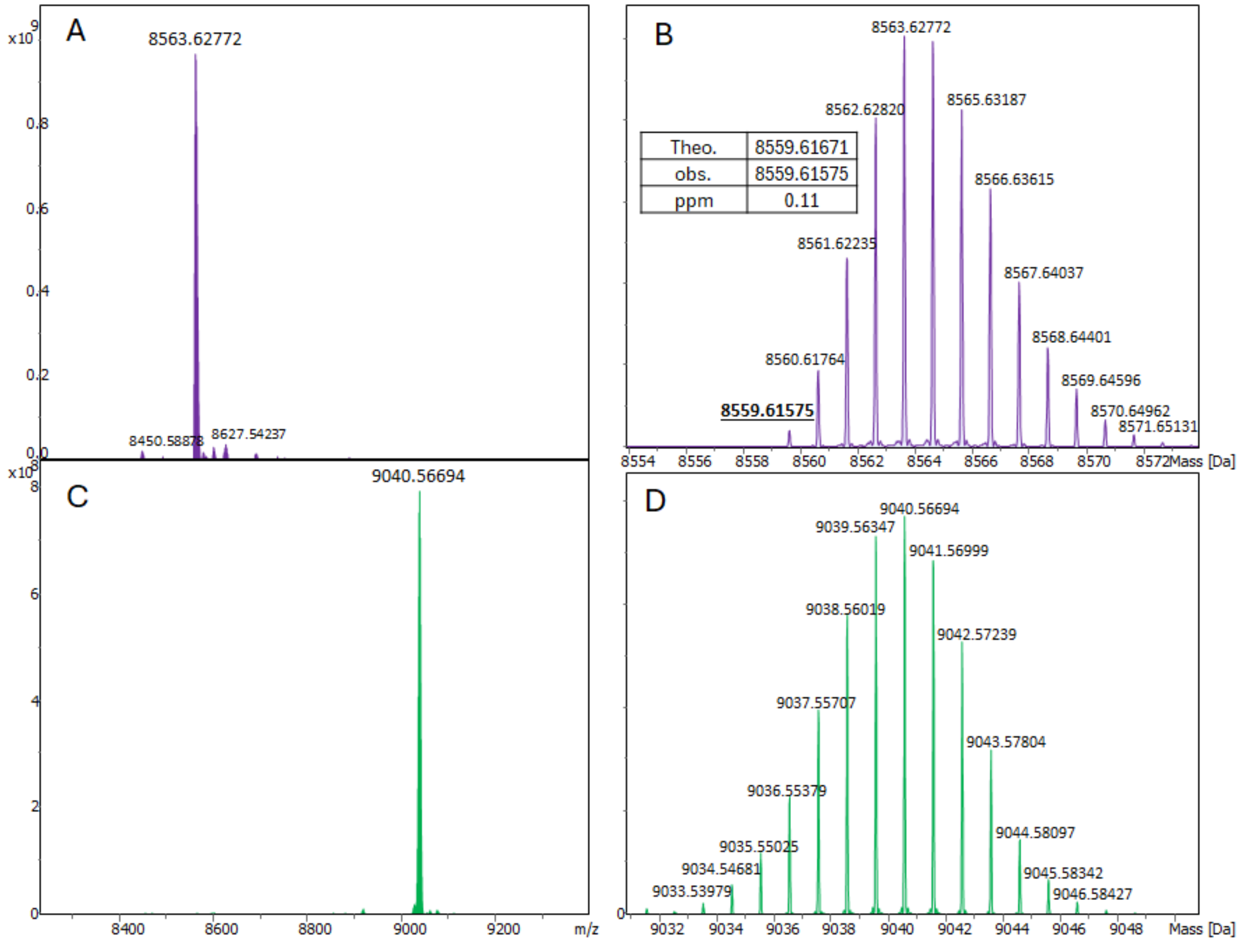

Figure S2: A) Deconvoluted spectrum of natural abundance ubiquitin. B) Zoomed view of deconvoluted natural abundance ubiquitin. The monoisotopic peak is bolded and underlined. C) Deconvoluted spectrum of uniformly [^{15}N ^{13}C] ubiquitin. D) Zoomed view of deconvoluted [^{15}N ^{13}C] Ubiquitin.

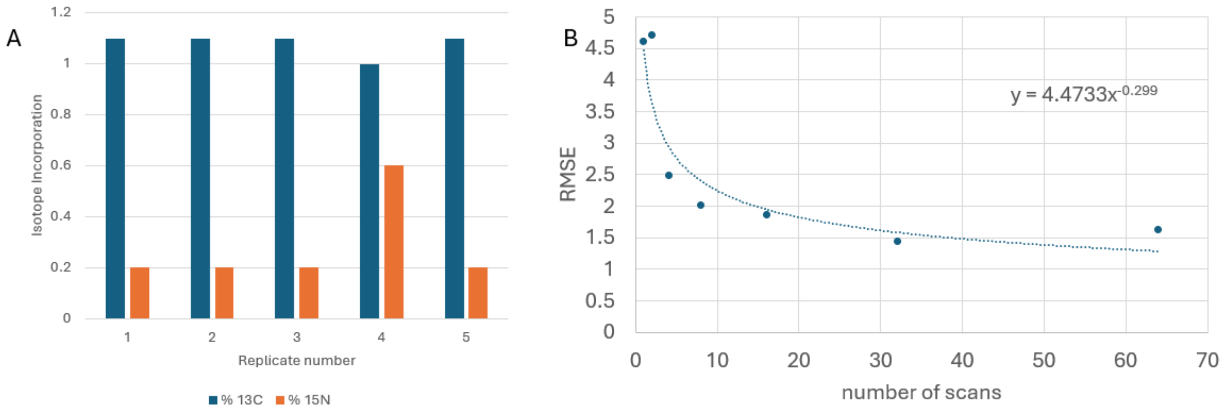

Figure S3: A) Replicate analysis of intact ubiquitin at natural abundance. Each spectrum was analyzed with a 1 ppm error tolerance. B) RMSE fit between simulated and experimental isotope patterns for intact ubiquitin as a function of number of scans.

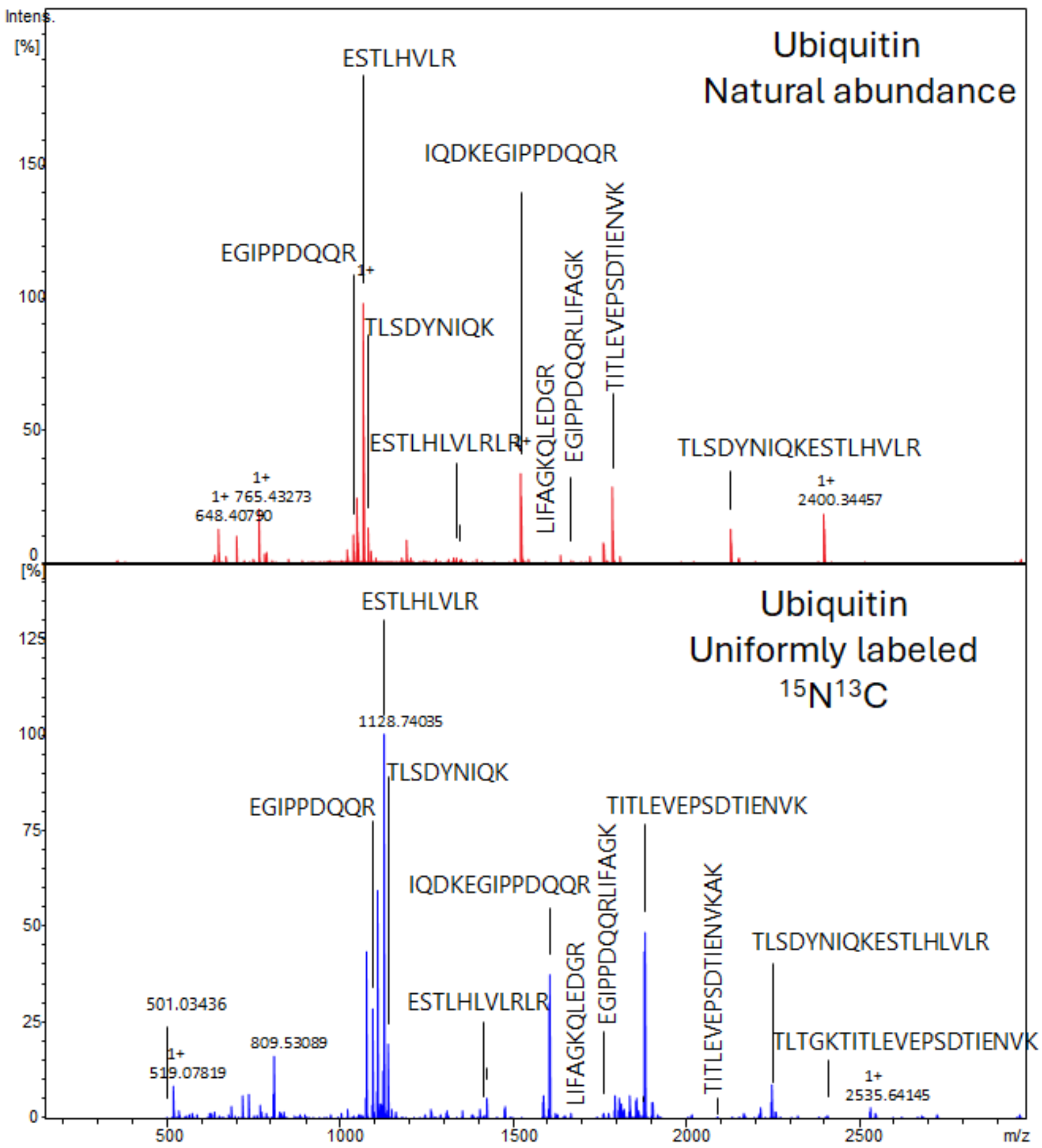

Figure S4: MALDI mass spectra of tryptic digests of natural abundance ubiquitin (top) and [15N 13C] ubiquitin (bottom).

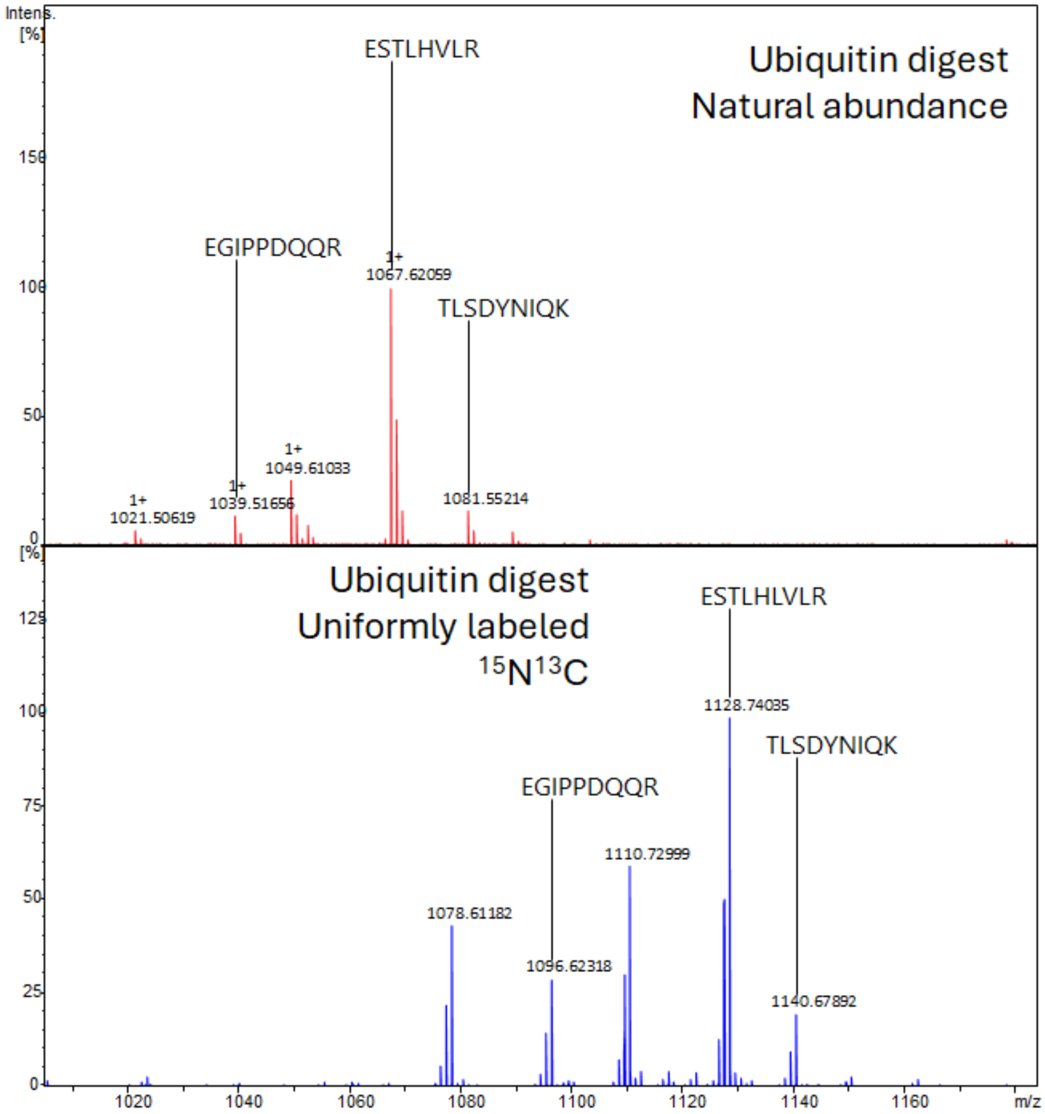

Figure S5: MALDI mass spectra of tryptic digests of natural abundance ubiquitin (top) and [15N 13C] ubiquitin (bottom) between 1010 – 1180 m/z.

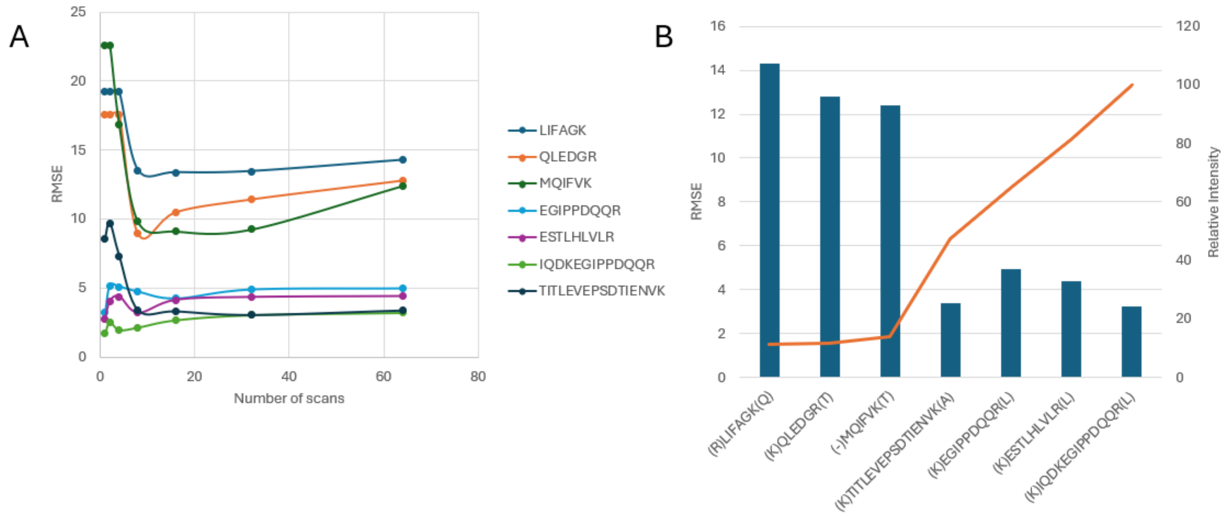

Figure S6: A) Effect of signal averaging on the RMSE scores for ubiquitin peptides at natural abundance. B) RMSE scores for ubiquitin peptides with 64 averaged scans. Peptides are arranged in order of their intensity (orange line).

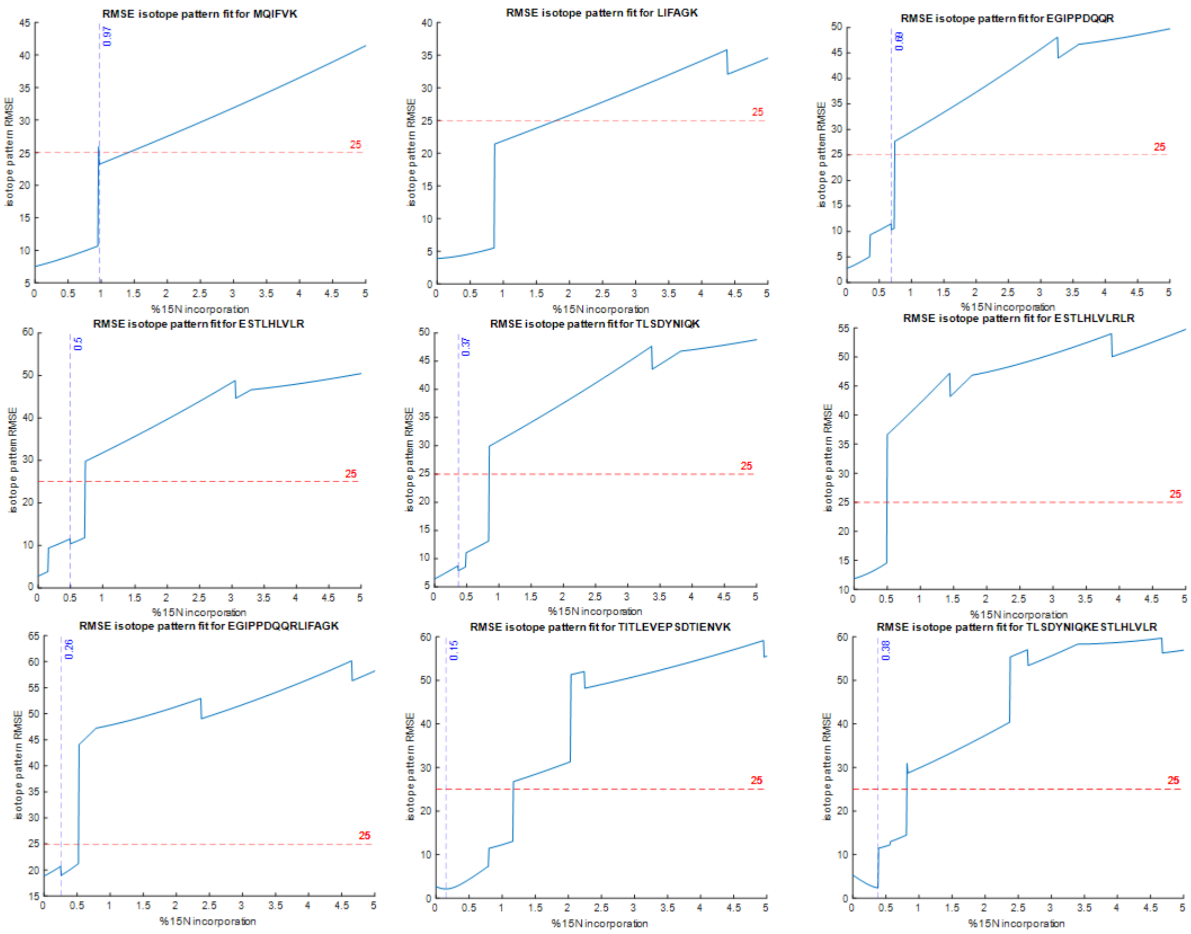

Figure S7: RMSE plots for the natural abundance ubiquitin digest treating only ^{15}N as an unknown.

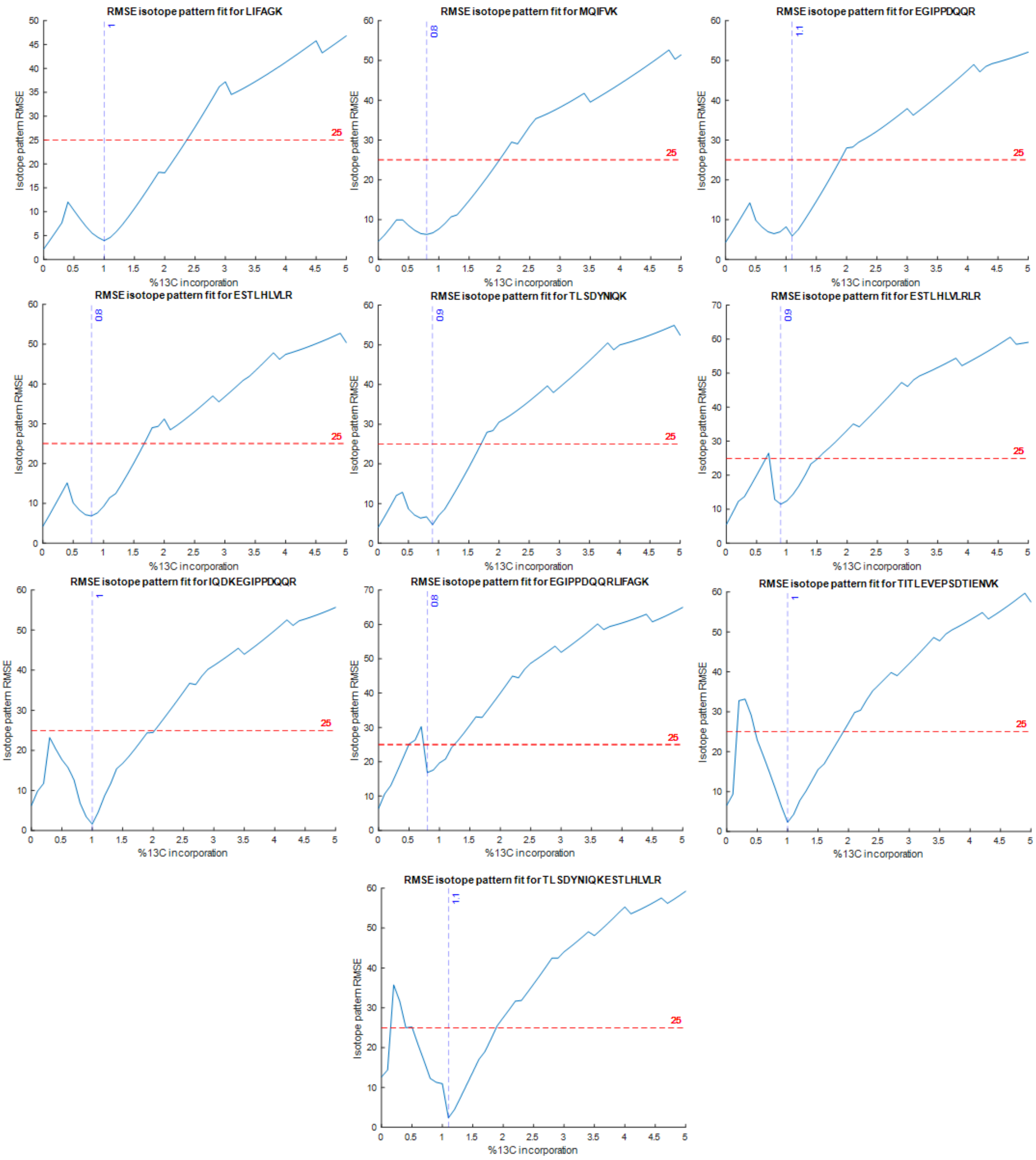

Figure S8: RMSE plots for the natural abundance ubiquitin digest treating only 13C as an unknown.

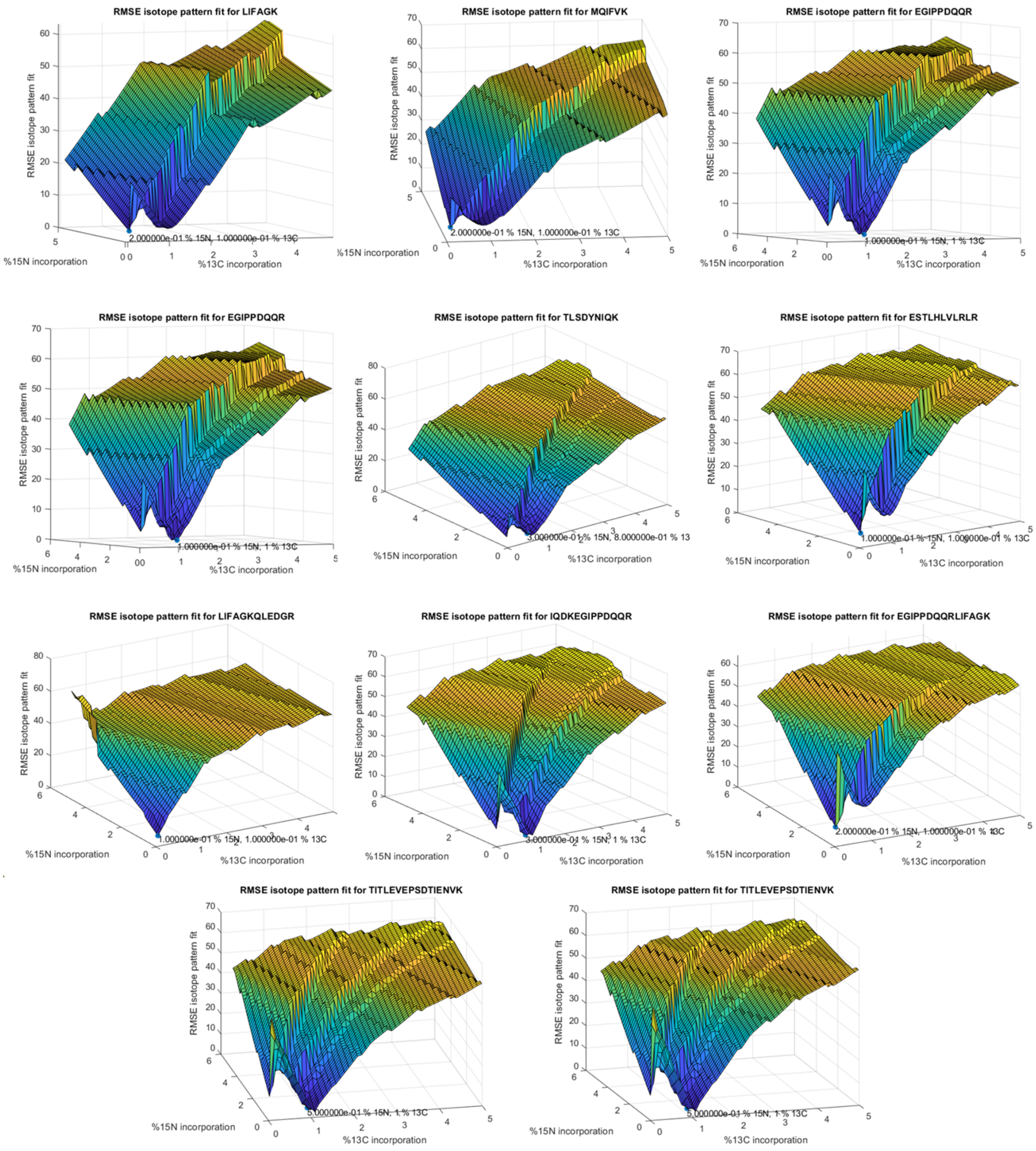

Figure S9: RMSE surface plots for natural abundance ubiquitin peptides . RMSE was calculated as a function of both ^{15}N and ^{13}C enrichment.

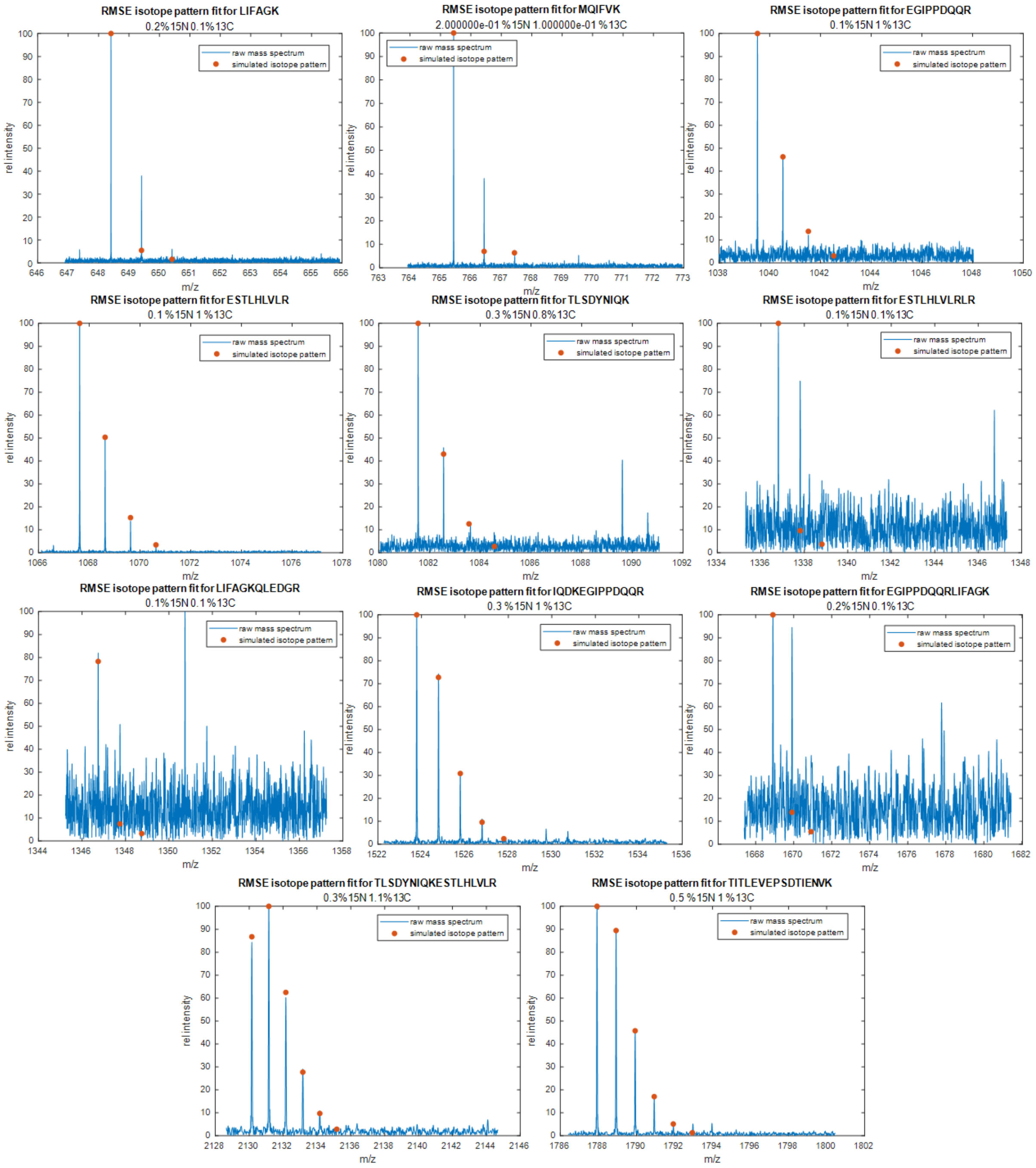

Figure S10: MALDI mass spectra of tryptic peptides of ubiquitin at natural abundance. Each spectrum is overlaid with an isotope pattern simulation (orange) that was determined the “best fit.”

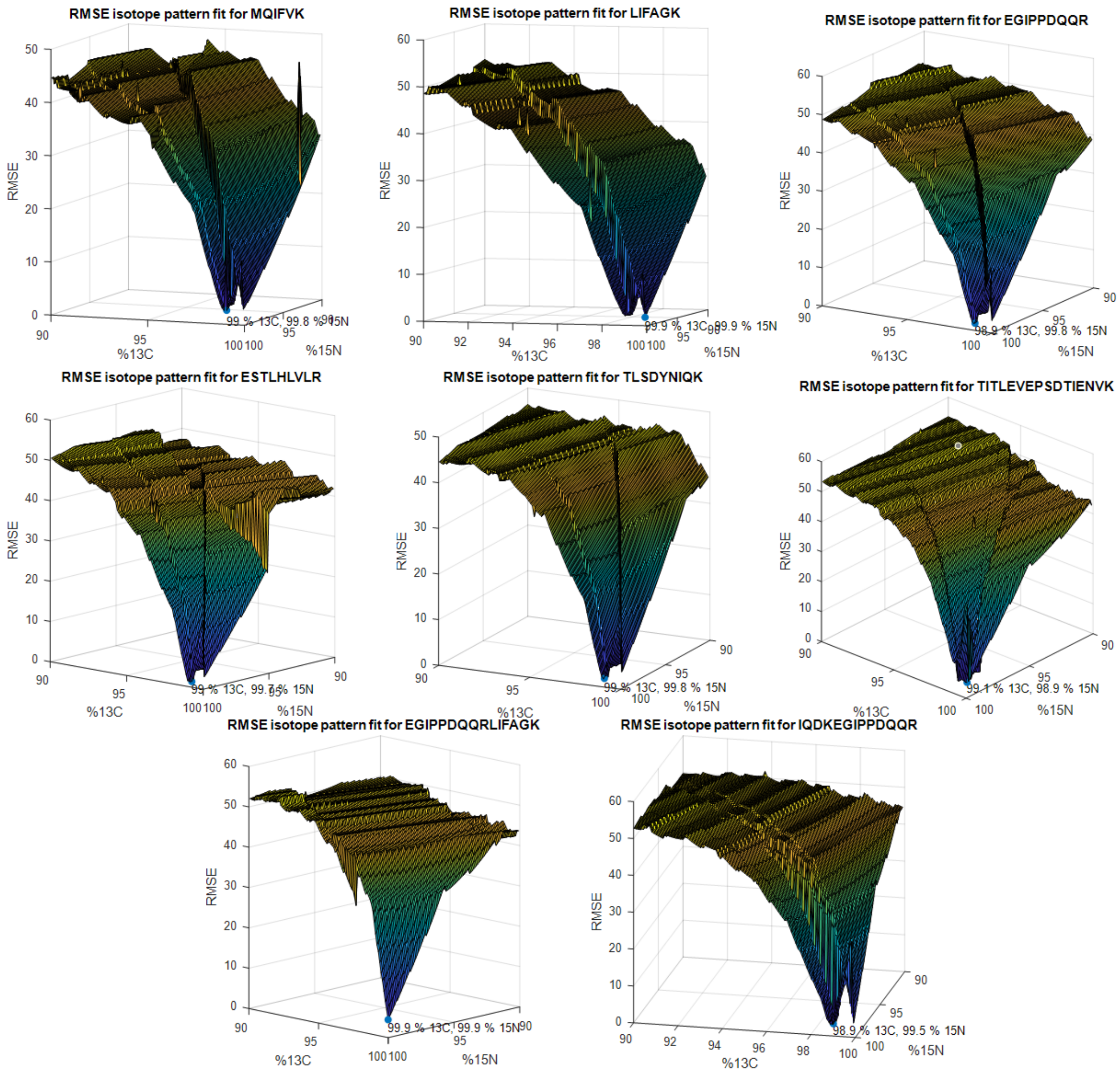

Figure S11: RMSE surface plots for [^{15}N ^{13}C] ubiquitin peptides . RMSE was calculated as a function of both 15N and 13C enrichment. High RMSE scores are shown in yellow and low RMSE scores are shown in blue.

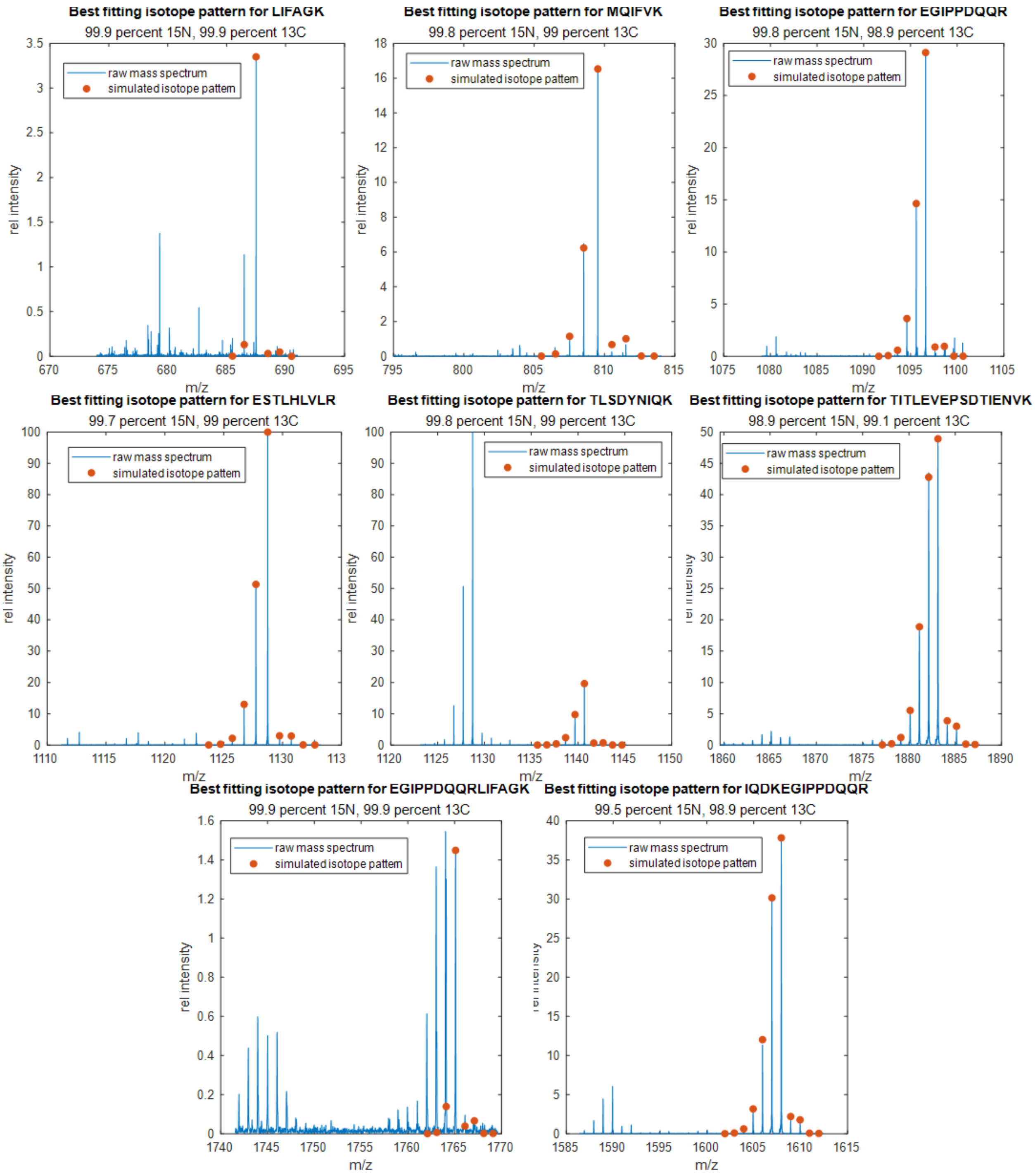

Figures S12: Mass spectra for [^{15}N ^{13}C] Ubiquitin peptides (blue) overlaid with the isotope pattern with the best fitting ^{15}N and ^{13}C abundance (orange).

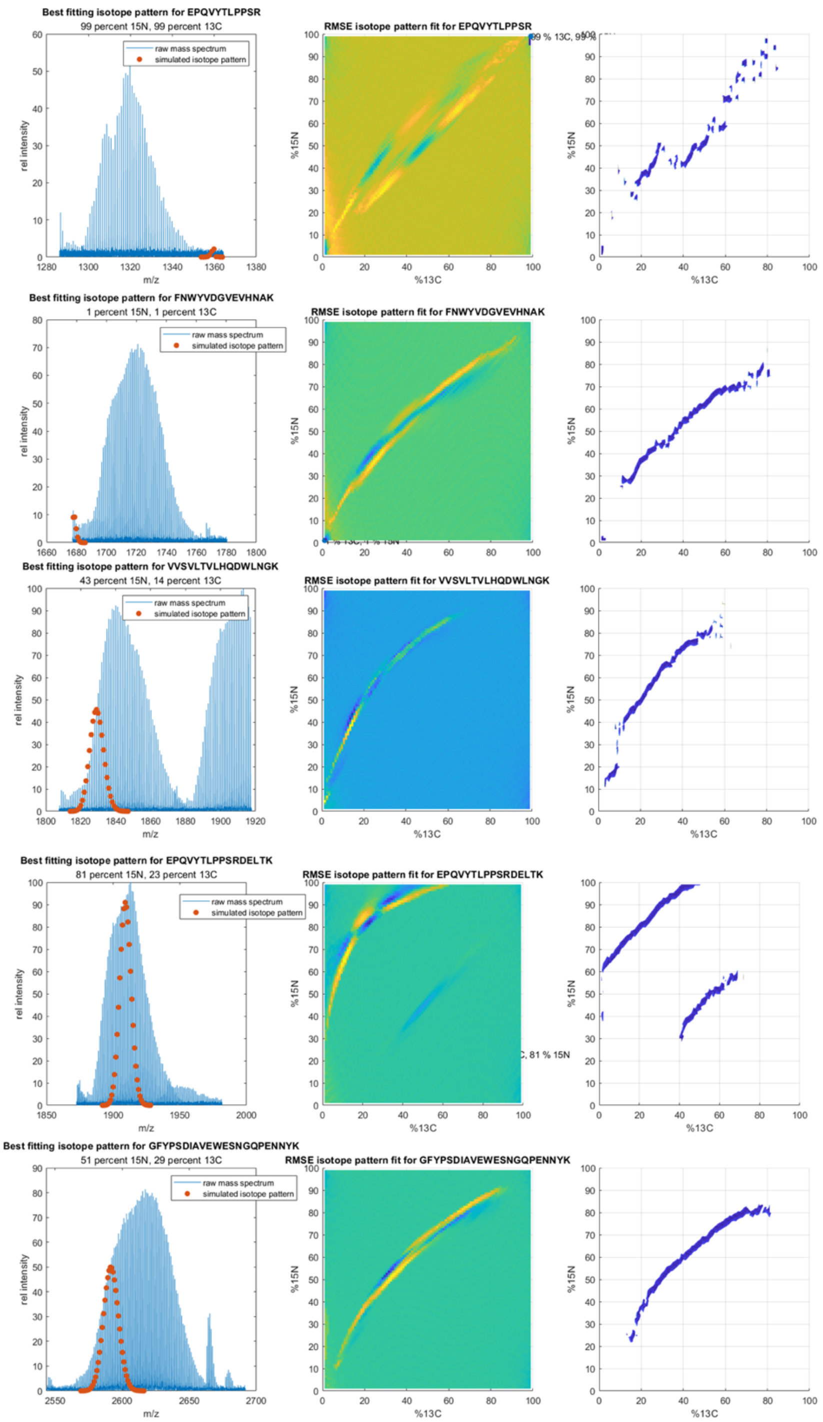

Figure S13: Two-dimensional grid searches for the [^{15}N ^{13}C] Fc domain with 5% unlabeled medium. Left: The mass spectra for each peptide along with their "best fitting" simulation are shown on the left. Middle: RMSE plots for the fit of each isotope pattern as a function of ^{15}N and ^{13}C. Good fits are shown in blue and bad fits are shown in yellow. Right: PPM error surface plot as a function of 15N and 13C. All ppm errors > 1 ppm were removed.

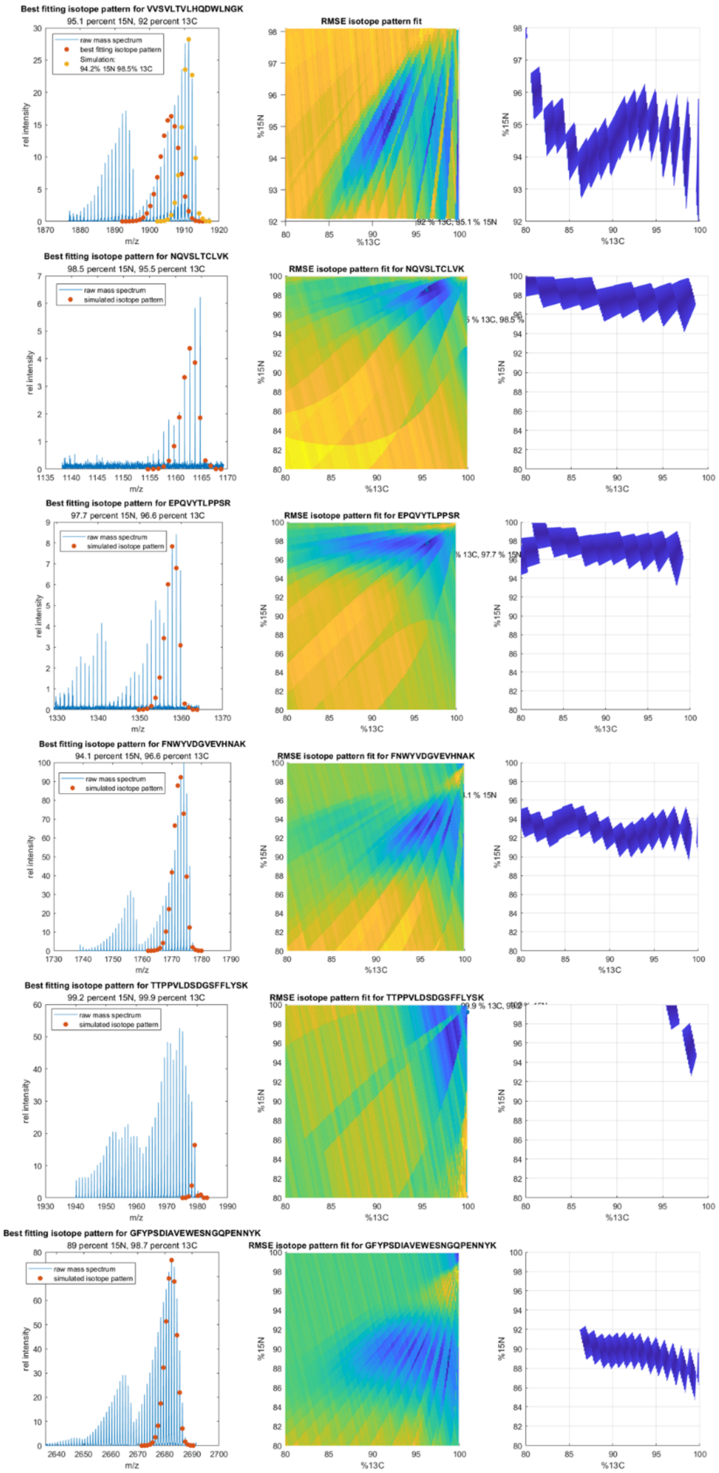

Figure S14: Two dimensional grid searches for the [^{15}N ^{13}C] Fc domain with 5% unlabeled medium. Left: The mass spectra for each peptide along with their "best fitting" simulation are shown on the left. Middle: RMSE plots for the fit of each isotope pattern as a function of ^{15}N and ^{13}C. Good fits are shown in blue and bad fits are shown in yellow. Right: PPM error surface plot as a function of 15N and 13C. All ppm errors > 1 ppm were removed.

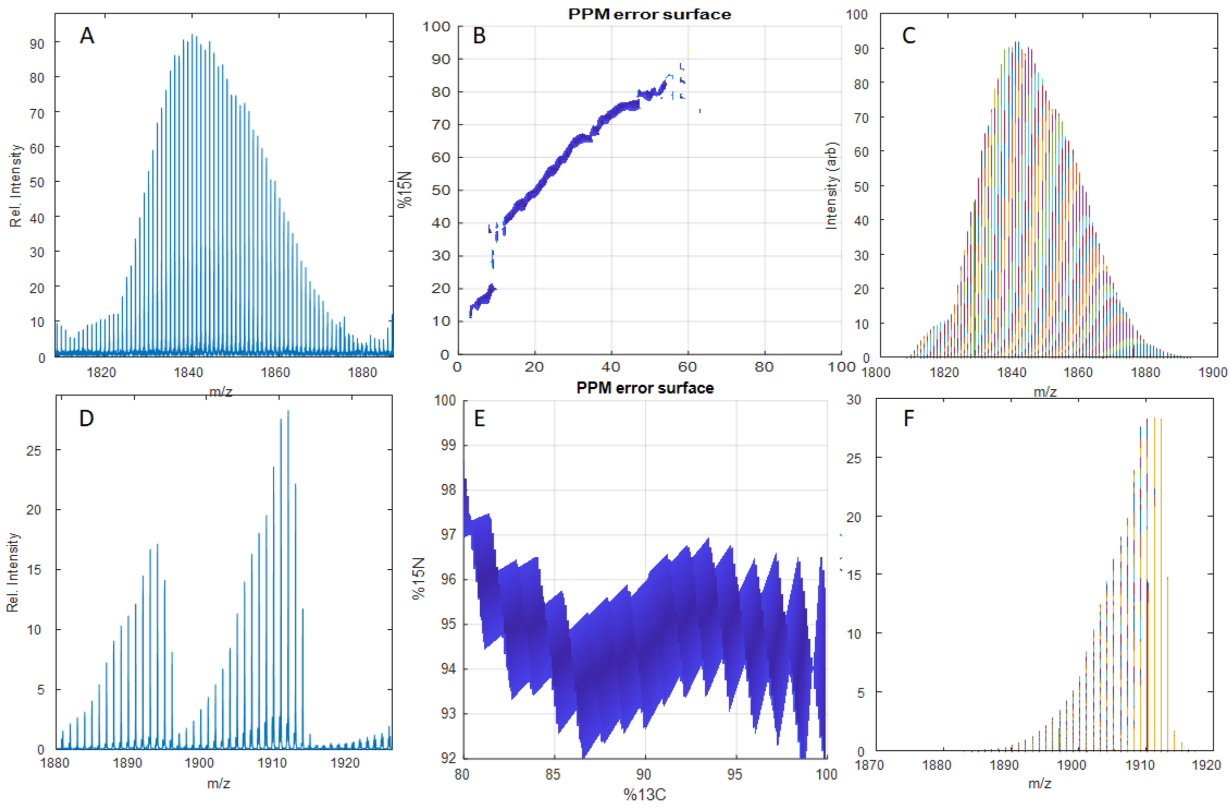

Figure S15: A) Mass spectrum for the 15N 13C labeeld peptide VVSVLTVLHQDWLNGK with 5% natural abundance medium. B) Ppm error surface for the most abundant peak in a simulated isotope distribution for the peptide in A as a function of ^{15}N and ^{13}C. The blue region represents ppm errors that were < 0.5 ppm. C) Overlaid isotope pattern simulations for VVSVLTVLHQDWLNGK with ^{15}N and ^{13}C incorporations that were determined from the minima in B. Simulations were generated in js-emass. D) Mass spectrum for the ^{15}N ^{13}C labeled peptide VVSVLTVLHQDWLNGK with homemade heavy isotope labeled yeast extract. E) Ppm error for the most abundant peak in a simulated isotope distribution for the peptide in A as a function of ^{15}N and ^{13}C. The blue region represents ppm errors that were < 1 ppm. F) Overlaid isotope pattern simulations for VVSVLTVLHQDWLNGK with ^{15}N and ^{13}C incorporations that were determined from E. Simulations were generated in js-emass.

Table S1: *Results for the replicate analysis of intact ubiquitin shown in figure S-3a.*

Replicate	% 15N	% 13C	RMSE	Theo max intensity m/z	obs max intensity m/z	ppm error
1	0.2	1.1	1.3896	8563.6287	8563.6281	0.073
2	0.2	1.1	1.4991	8563.6287	8563.6283	0.0496
3	0.2	1.1	1.4678	8563.6287	8563.6284	0.0286
4	0.6	1	1.4566	8564.6289	8564.6318	0.3336
5	0.2	1.1	1.3997	8563.6287	8563.6285	0.0274
**AVG**	**0.28**	**1.08**				
**STDEV**	**0.179**	**0.045**				
**RSTDEV**	**0.639**	**0.041**				

Table S2: *MS-Fit results (Protein Prospector) for a digest of natural abundance ubiquitin shown in Figure S4.*

1. 14/62 matches (22%).
Acc. #: 1 **Species:** UNREADABLE **Name:**
Version: 1
Index: 1 **MW:** 8565 Da **pI:** 6.6

m/z Submitted	MH^+ Matched	Intensity	Delta ppm	Modifications	Start	End	Missed Cleavages	Sequence
648.4080	648.4079	8388217.0	0.180		43	48	0	(R)LIFAGK(Q)
700.3260	700.3260	6481363.0	-0.0777	1Gln->pyro-Glu	49	54	0	(K)QLEDGR(T)
765.4329	765.4328	13017550.0	0.146		1	6	0	(-)MQIFVK(T)
781.4276	781.4277	2449354.0	-0.106	1Oxidation	1	6	0	(-)MQIFVK(T)
1039.5168	1039.5167	6795484.0	0.0590		34	42	0	(K)EGIPPDQQR(L)
1067.6209	1067.6208	58711852.0	0.103		64	72	0	(K)ESTLHLVLR(L)
1081.5524	1081.5524	7878683.0	0.00414		55	63	0	(R)TLSDYNIQK(E)
1336.8059	1336.8059	1636436.0	-0.0111		64	74	1	(K)ESTLHLVLRLR(G)
1346.7442	1346.7427	878379.0	1.16		43	54	1	(R)LIFAGKQLEDGR(T)
1523.7815	1523.7812	20165356.0	0.186		30	42	1	(K)IQDKEGIPPDQQR(L)
1668.9077	1668.9068	844730.0	0.563		34	48	1	(K)EGIPPDQQRLIFAGK(Q)
1762.8617	1762.8606	5163382.0	0.602	1Gln->pyro-Glu	49	63	1	(K)QLEDGRTLSDYNIQK(E)
1787.9275	1787.9273	17133534.0	0.122		12	27	0	(K)TITLEVEPSDTIENVK(A)
2130.1555	2130.1553	8137577.0	0.0966		55	72	1	(R)TLSDYNIQKESTLHLVLR(L)

Num Unmatched Masses: **48**
Search for disulfide linked peptides.
Do a non-specific cleavage search.
Search for another component.

The matched peptides cover **88.2%** (67/76AA's) of the protein.
Coverage Map for This Hit (MS-Digest index #): 1

Table S3: Isotopic abundance results for ubiquitin peptides treating only ^{15}N as an unknown.

Peptide Sequence	%15N incorporation	RMSE score	Theo max int. m/z	Obs max int. m/z	ppm error
EGIPPDQQR	0.69	-10.362	1039.517	1039.517	0.637
ESTLHLVLR	0.5	-10.39	1067.621	1067.621	0.659
TLSDYNIQK	0.37	-7.8848	1081.553	1081.552	0.739
IQDKEGIPPDQQR	0.09	-1.4602	1523.782	1523.782	0.111
EGIPPDQQRLIFAGK	0.26	-18.98	1668.907	1668.908	0.399
TITLEVEPSDTIENVK	0.15	-2.1323	1787.928	1787.927	0.243
TLSDYNIQKESTLHLVLR	0.38	-2.3645	2131.158	2131.159	0.176
AVERAGE	0.349				
STDEV	0.206				
RSTDEV	59.153				

Table S4: Isotopic abundance results for ubiquitin peptides treating only ^{13}C as an unknown.

Peptide Sequence	%13C incorporation	RMSE score	Theo max int. m/z	Obs max int. m/z	ppm error
LIFAGK	1	3.9285	648.4084	648.4079	0.8437
MQIFVK	0.8	6.2722	765.4333	765.4327	0.7207
EGIPPDQQR	1.1	5.8971	1039.5172	1039.5166	0.6371
ESTLHLVLR	0.8	6.8375	1067.6213	1067.6206	0.6589
TLSDYNIQK	0.9	4.6684	1081.5529	1081.5521	0.7388
ESTLHLVLRLR	0.9	11.4749	1336.8065	1336.8058	0.4925
IQDKEGIPPDQQR	1	1.5813	1523.7818	1523.7816	0.1114
EGIPPDQQRLIFAGK	0.8	16.8608	1668.9073	1668.908	0.3987
TITLEVEPSDTIENVK	1	2.319	1787.9278	1787.9274	0.2433
TLSDYNIQKESTLHLVLR	1.1	2.3541	2131.1584	2131.1588	0.1633
AVERAGE	0.94				
RSTDEV	12.49				

Table S5: Isotopic abundance results for ubiquitin peptides treating both ^{15}N and ^{13}C as unknowns.

Peptide Sequence	%15N incorporation	%13C incorporation	RMSE score	Theo max int. m/z	Obs max int. m/z	ppm error
LIFAGK	0.2	0.1	3.2937	648.4084	648.4079	0.8437
MQIFVK	0.2	0.1	5.4749	765.4333	765.4327	0.7207
EGIPPDQQR	0.1	1	2.2996	1039.5172	1039.5166	0.6371
ESTLHLVLR	0.1	1	2.017	1067.6213	1067.6206	0.6589
TLSDYNIQK	0.3	0.8	3.1345	1081.5529	1081.5521	0.7388
ESTLHLVLRLR	0.1	0.1	5.9614	1336.8065	1336.8058	0.4925
LIFAGKQLEDGR	0.1	0.1	6.0004	1346.7432	1346.743	0.1333
IQDKEGIPPDQQR	0.3	1	1.4833	1523.7818	1523.7816	0.1114
EGIPPDQQRLIFAGK	0.2	0.1	8.6485	1668.9073	1668.908	0.3987
TITLEVEPSDTIENVK	0.5	1	2.1976	1787.9278	1787.9274	0.2433
TLSDYNIQKESTLHLVLR	0.3	1.1	2.3072	2131.1585	2131.1588	0.123
AVERAGE	0.218	0.581				
RSTDEV	57.31	80.18				
